# Characterization of tumor microenvironment and tumor immunology based on the double-stranded RNA-binding protein related genes in cervical cancer

**DOI:** 10.1186/s12967-023-04505-9

**Published:** 2023-09-21

**Authors:** Jin Li, Chong Wan, Xiaoqi Li, Chenlian Quan, Xiaoqiu Li, Xiaohua Wu

**Affiliations:** 1 Department of Gynecologic Oncology, Fudan University Shanghai Cancer Center, Fudan University, No. 270 Dong’an Road, Shanghai, 200032 China; 2 Department of Pathology, Fudan University Shanghai Cancer Center, Fudan University, Shanghai, 200032 China; 3 Department of Oncology, Shanghai Medical College, Fudan University, Shanghai, 200032 China; 4 Precision Medicine Center, Yangtze Delta Region Institute of Tsinghua University, Jiaxing, China

**Keywords:** Cervical cancer, dsRBP, Risk model, Tumor microenvironment, Immunotherapy, Chemotherapy

## Abstract

**Background:**

Cervical cancer is one of the most common gynecological cancers threatening women’s health worldwide. Double-stranded RNA-binding proteins (dsRBPs) regulate innate immunity and are therefore believed to be involved in virus-related malignancies, however, their role in cervical cancer is not well known.

**Methods:**

We performed RNA-seq of tumor samples from cervical cancer patients in local cohort and also assessed the RNA-seq and clinical data derived from public datasets. By using single sample Gene Set Enrichment Analysis (ssGSEA) and univariate Cox analysis, patients were stratified into distinct dsRBP clusters. Stepwise Cox and CoxBoost were performed to construct a risk model based on optimal dsRBPs clusters-related differentially expressed genes (DEGs), and GSE44001 and CGCI-HTMCP-CC were employed as two external validation cohorts. Single cell RNA sequencing data from GSE168652 and Scissor algorithm were applied to evaluated the signature-related cell population.

**Results:**

The expression of dsRBP features was found to be associated with HPV infection and carcinogenesis in CESC. However, only Adenosine deaminases acting on RNA (ADAR) and Dicer, Drosha, and Argonautes (DDR) exhibited significant correlations with the overall survival (OS) of CESC patients. Based on these findings, CESC patients were divided into three dsRBP clusters. Cluster 3 showed superior OS but lower levels of ADAR and DDR. Additionally, Cluster 3 demonstrated enhanced innate immunity, with significantly higher activity in cancer immunity cycles, immune scores, and levels of tumor-infiltrating immune cells, particularly CD8+ T cells. Furthermore, a risk model based on nine dsRBP cluster-related DEGs was established. The accuracy of survival prediction for 1 to 5 years was consistently above 0.78, and this model’s robust predictive capacity was confirmed by two external validation sets. The low-risk group exhibited significantly higher levels of immune checkpoints, such as PDCD1 and CTLA4, as well as a higher abundance of CD8+ T cells. Analysis of single-cell sequencing data revealed a significant association between the dsRBP signature and glycolysis. Importantly, low-risk patients showed improved OS and a higher response rate to immunotherapy, along with enduring clinical benefits from concurrent chemoradiotherapy.

**Conclusions:**

dsRBP played a crucial role in the regulation of prognosis and tumor immunology in cervical cancer, and its prognostic signature provides a strategy for risk stratification and immunotherapy evaluation.

**Supplementary Information:**

The online version contains supplementary material available at 10.1186/s12967-023-04505-9.

## Introduction

Cervical cancer is the fourth leading cause of female cancers globally, resulting in approximately 600,000 new cases and more than 300,000 deaths in 2020 [1]. Numerous studies have established a strong association between cervical cancer and high-risk human papillomavirus (hrHPV) infection, particularly hrHPV types 16 and 18. It has been observed that hrHPV infection can lead to the development of malignant tumors through the progression of precancerous lesions [2, 3]. Notable progress has been achieved in the screening, diagnosis, and treatment of cervical cancer. Primary hrHPV testing and cervical cytology screening are among the significant advancements that have contributed to a significant decrease in the incidence and mortality of cervical cancer among individuals aged 21 to 65 [4]. In the management of cervical cancer, radical hysterectomy and chemoradiation remain the primary treatment approaches. Additionally, it has been observed that the chemotherapy drug bevacizumab provides survival benefits for patients with advanced disease [5]. Nonetheless, these conventional treatments have been associated with adverse effects, and despite advancements, metastatic cervical cancer is still considered incurable [6]. Therefore, there is an urgent need to identify reliable biomarkers for cervical cancer that can facilitate more effective clinical management.

Double-stranded RNA-binding protein (dsRBP) is defined to identify the universal RNA double-stranded body through a highly conserved double-stranded RNA binding domain (dsRBD). In eukaryotes, dsRBP is mainly responsible for the RNA editing, stability maintenance, and functionality of RNA [7]. There are mainly seven subtypes in dsRBP, including RIG-i-like receptor (RLR), protein kinase R (PKR), oligoadenylate synthases (OAS) and RNase L, adenosine deaminases acting on RNA (ADAR), Dicer, Drosha and Argonautes (DDR), PACT and TRBP, helicase, which are all involved in the antiviral innate immunity of mammalian [8]. Studies have shown that dsRBPs are related to the carcinogenesis and progression in various types of cancers. For instance, RIG-I, a member of the RLR family, has a strong association with hepatocellular carcinoma, primarily impacting the biological activities of matrix metalloproteinase-9 [9]. As for PKR, recent research has indicated that its overexpression is linked to poor survival rates among breast cancer patients [10]. Overexpression of ADAR1 has been significantly associated with hematological malignancies [11]. Moreover, MiR-346 has been found to promote up-regulation of Ago2 protein expression, thereby enhancing the proliferation and migration of cervical cancer cells [12]. It has been observed that the induction of dsRNA stress-encoded neoantigens can enhance interferon signaling and tumor immunogenicity [13]. Moreover, the activation of dsRNA stress-type 1 interferon signaling has been found to stimulate anti-tumor T cell immunity and inhibit tumor growth, making it a potential approach to increasing the sensitivity of immune checkpoints inhibitors (ICIs) in poorly immunogenic tumors [14]. Meanwhile, it has been demonstrated that the ablation of KMT2D, a common occurrence in multiple types of cancer, has the potential to activate dsRNA-interferon signaling, leading to an enhanced immunotherapeutic efficacy [15]. Despite the aforementioned findings, to date, there still lacks study that comprehensively investigated the potential prognostic or immunogenic functions of the seven subtypes of dsRBPs in cervical cancer.

Our research was implemented to determine the heterogeneity in the expression feature of dsRBPs and the correlation between dsRBPs and the clinical characteristics, genomic profiles, immune cell infiltration of cervical cancer. Based on the ssGSEA and univariate Cox analysis, we obtained the dsRBPs that were associated with the OS of cervical cancer, and clustered the TCGA-cervical squamous cell carcinoma (CESC) dataset into distinct molecular clusters. Besides, the survival curves, genetic mutation analysis, TME landscape, and immune features among the different molecular patterns were investigated. Eventually, we established a risk model on basis of cluster-associated DEGs, which can further identify the prognostic role and therapeutic response of dsRBPs in cervical cancer.

## Materials and methods

### Data download and preparation

As a training cohort, 304 patients with cervical cancer were collected from The Cancer Genome Atlas (TCGA) database (http://xena.ucsc.edu/), along with their RNA expression details and clinical information. The HPV infection status of 178 samples from the TCGA-CESC cohort was obtained from a publicly available study [6]. The GSE44001 from Gene Expression Omnibus (GEO) database (https://www.ncbi.nlm.nih.gov/geo/), CGCI-HTMCP-CC from Genomic Data Commons Data Portal (GDC) database (https://portal.gdc.cancer.gov/) and transcriptomic data of cervical patients from local cohorts were set as independent validation cohorts. Additionally, the immunotherapy dataset IMvigor210 was downloaded from IMvigor210CoreBiologies (http://research-pub.gene.com/IMvigor210CoreBiologies), and the chemotherapy dataset GSE168009 was obtained from the GEO database as the source to evaluate the association between identified signature and treatment efficacy. Next, the seven dsRBP subtypes from the previous study [8] were utilized as the keywords in the GeneCard (https://www.genecards.org/), resulting in a collection of 35 dsRBP genes (Additional file 2: Table S1).

### RNA sequencing in the local cervical cancer patients

Tumor and matched normal tissues were collected from 15 cervical cancer patients in the Fudan university shanghai cancer center (FUSCC) cohort to perform RNA-seq. This study was approved by the Ethics Committee of FUSCC and written informed consent was obtained from all the patients. Before RNA extraction, the tissue was evaluated for tumor cell content, and only those with a tumor purity of at least 20% based on histopathological analysis were eligible for RNA extraction and sequencing. Total RNA from each sample was collected using a FastPure® Cell/Tissue Total RNA Isolation Kit V2 (Vazyme, Jiangsu, China), and the RNA concentration and RNA integrity number (RIN) were measured using a Qubit (Thermo Fisher Scientific, MA, United States) and an Agilent 2100 bioanalyzer (Agilent Technologies, CA, United States), respectively. Library construction was performed using the NEBNext® Ultra™ RNA Library Prep Kit for Illumina® Kit (NEB, MA, United States) and sequenced on the Illumina Novaseq-6000 system (Illumina, MA, United States).

### Gene set enrichment analysis (ssGSEA) and consensus clustering of dsRBPs

We performed single sample ssGSEA to quantitatively illustrate enrichment scores of the seven dsRBPs gene sets using the Gene Set Variation Analysis (GSVA) R package [16]. In addition, based on the ssGSEA scores, we employed consensus clustering to identify different dsRBP-related patterns in the TCGA-CESC cohort using the k-means algorithms [17] with the R package “ConsensuClusterPlus” [18]. The cumulative distribution function (CDF) was used to identify the final number of clusters. Furthermore, Kaplan–Meier analysis was applied to explore the overall survival (OS) of distinct clusters with “survival” and “survminer” R packages [19], and principal components analysis (PCA) was carried out with “FactoMineR” package.

### Genomic characteristics

The somatic mutation profile of cervical cancer was identified by mutation annotation format (MAF) file with the R package “maftools” [20]. Fisher’s test was applied to investigate the frequently mutated genes. We obtained copy number variation (CNV) data from TCGA database and employed GISTIC2.0 to identify the amplification and deletion regions in high- and low-risk groups.

### TME landscape

XCELL and CIBERSORT were employed to uncover the immune infiltrating abundance of tumor-infiltrating immune cells (TIICs) in the TME. Moreover, we calculated the stromal score, immune score, and ESTIMATE score according to the ESTIMATE algorithm [21] using the “estimate” R package.

### Immunological features evaluation

Accordingly, we investigated the potential association between three clusters and ICIs-related genes, and the expression level of HLA. In addition, we collected multiple immune signatures to compare the differences between different molecular patterns according to Kobayashi (glycolysis, IFN-γ response, inhibitory cells MDSCs, inhibitory cells Tregs, inhibitory molecules, innate immunity, priming activation, proliferation, recognition of tumor cells, and T cells) [22] and Bagaev (angiogenesis, anti-tumor microenvironment, antigen presentation, B cells, CAF, checkpoint inhibition, cytotoxic T and NK cells, granulocytes, MDSC, Treg, tumor features, tumor promotive immune infiltrate) [23].

### Identification of differentially expressed genes (DEGs)

According to “limma” R package [24] with criteria of |log2-fold change (FC)| ≥ 1 and p-value < 0.01, we obtain the DEGs between the identified clusters, which were visualized using volcano plots by the “ggplot2” R package [25]. Subsequently, univariate Cox analysis was applied to screen the prognostic cluster-associated DEGs with a p-value < 0.01. Moreover, we screened out optimal DEGs according to whether they were differentially expressed between cancer and normal tissues using the “limma” R package based on prognostic cluster-associated DEGs, and the cutoff criterion was set as p-value < 0.01. Moreover, Kyoto Encyclopedia of Genes and Genomes (KEGG) [26] and gene set enrichment analysis were performed to identify the biological functions of cluster-associated DEGs, and the hallmark pathways of GSEA analysis were extracted from Molecular Signatures Database (MsigDB, http://www.gsea-msigdb.org/gsea/downloads.jsp).

### Construction and verification of risk model

According to the optimal DEGs, we performed 42 combinations of 6 machine learning algorithms, including Ridge, CoxBoost, elastic network (Enet), least absolute shrinkage and selection operator (LASSO) regression analysis, stepwise Cox, and random survival forest (RSF) to filtrate the most valuable risk model. Accordingly, the risk model was constructed depending on the stepwise Cox and CoxBoost with the highest C-index. Stepwise Cox was applied to filtrate the most valuable DEGs, and CoxBoost was used to screen the most reliable model. Then, the patients in the TCGA-CESC database were assigned to the high-risk group and low-risk group based on the median risk score. Kaplan–Meier curves were analyzed, and the receiver operating characteristic (ROC) curves at 12, 24, 36, 48, and 60 months were performed with the “timeROC” R package [27]. GSE44001 and CGCI-HTMCP-CC datasets were extracted as two external validation cohorts to further evaluate the reliability of the risk model. Furthermore, survival analysis was implemented to verify the characteristics of the = high-and low-risk groups stratified by clinical parameters, including age (≤ 60/> 60), T stage (T1–2/T3–4), N stage (N0/N1+), M stage (M0/M1), and pathological stage (stage I–II/stage III–IV).

### Single cell RNA sequecing acquisition and SCISSOR analysis

The single-cell RNA sequencing data for the GSE168652 dataset [28] was obtained from the TISCH website [29]. The cell-type annotations were provided by combining the results from the original study with adjustments made using cell markers and the InferCNV algorithm employed by the TISCH study. We combined single cell data and TCGA bulk expression data using the scissor (2.0.0) method [30] to investigate cell subpopulations connected to risk groups. First, we classified TCGA-CESC patients into high-risk and low-risk groups, and then we used the scissor approach in conjunction with logistic regression to extract the most relevant cell populations with regard to risk group. We separated these cell groups into scissor-positive and scissor-negative categories, and finally, we identified differentially expressed genes using the “seurat package” (4.3.0) in R. In our current study, the term “scissor+ cells” refers to malignancy cells that exhibit a phenotype consistent with a high-dsRBP risk score in the bulk sequencing data, while “scissor− cells” refer to malignancy cells that exhibit a phenotype similar to the low-dsRBP risk score type.

### Immunotherapy response of risk model

Tumor immune dysfunction and exclusion (TIDE) analysis was performed to identify the response of immune checkpoint inhibitors, which has been proved as a excellent immunotherapy predictive biomakrer [31]. T cell dysfunction score, T cell exclusion score, and TIDE score were downloaded from the TIDE website (http://tide.dfci.harvard.edu). Besides, immunophenoscore (IPS) contains four types, such as immunosuppressive cells, immunomodulators, effector cells and MHC molecules, were reported to be a powerful biomarker of immunotherapy response to anti-PD-1 and anti-CTLA-4 therapy [32]. The IPS of cervical cancer patients were collected from the Cancer Immunome Atlas (TCIA, https://tcia.at/home) database. Moreover, the IMvigor210 dataset with anti-PD-L1 therapy was implemented to assess the immunotherapy value of the risk score using the “IMvigor210CoreBiologies” R package [33].

### Chemotherapy prediction

Based on the Genomics of Drug Sensitivity in Cancer (GDSC) database, we calculated the half-maximum inhibitory concentration (IC50) of cervical cancer patients using R package “pRRophetic” [34]. Furthermore, we applied the GSE168009 dataset with the concurrent chemoradiotherapy (CCRT) to evaluate the chemotherapy response of risk score.

### Statistical analysis

Kaplan–Meier plots and log-rank tests were employed to compare the survival ability of different groups. R software and its related software package (v.4.1.2) were utilized to analyze and process data. Continuous data processing was applied according to Wilcoxon's test. All tests were two-way and the p < 0.05 was performed in all of the analytic approached, suggesting that there was considered statistically significant.

## Results

### Characteristic the expression patterns of dsRBPs in cervical cancer

Firstly, we analyzed the expression features related to the HPV infection status in CESC patients. In comparison to HPV-negative CESC patients (n = 9), HPV-positive CESC patients in the TCGA-CESC cohort showed significantly lower expression levels of DDR and PACT_TRBP, but higher expression levels of OAS_RNAseL and RLR (Additional file 1: Fig. S1A). This trend remained consistent when we classified HPV-positive patients based on hierarchical HPV calls or clades (Additional file 1: Fig. S1B, C). However, we did not observe any significant differences in any of the dsRBP subtypes between CESC patients with (n = 141) or without (n = 37) HPV DNA integration (Additional file 1: Fig. S1D).

**Fig. 1:**
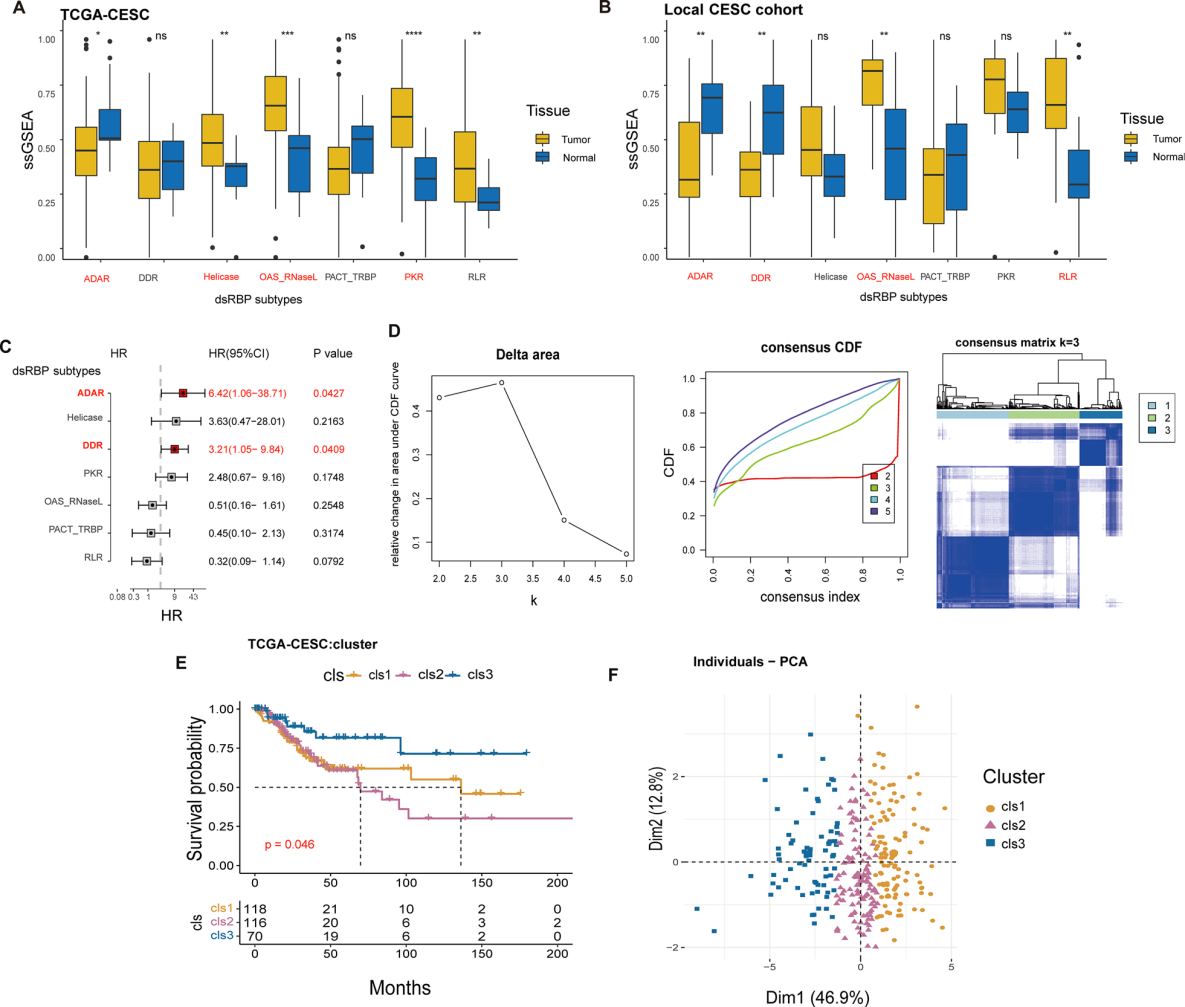
Identification of dsRBPs expression patterns in cervical cancer. Expression levels of dsRBPs in tumor (n = 307) and normal tissues (n = 3) in TCGA-CESC cohort (**A**) and FUSCC cohort (normal, n = 15; cancer, n = 15) (**B**). **C** The forest plot depicted the dsRBPs that were correlated with the OS of cervical cancer. **D** Consensus clustering of ADAR and DDR in the TCGA-CESC cohort. **E** Kaplan–Meier survival curve of the differences in the three clusters regarding OS (cluster 1, n = 118; cluster 2, n = 116; cluster 3, n = 70). **F** PCA of three clusters. dsRBPs: double-stranded RNA-binding proteins, *OS* overall survival, *ADAR* adenosine deaminases acting on RNA, *DDR* Dicer, Drosha, and Argonautes, *PCA* principal components analysis. *p < 0.05; ** p < 0.01; ***p < 0.001; ****p < 0.0001; ns: nonsignificant

In both TCGA-CESC and FUSCC cohorts (Fig. 1A, B), the expression pattern of dsRBPs-related subtypes or genes is consistent: ADAR and DDR subtypes are significantly downregulated in tumor tissues; whereas helicase, OAS RNAseL, PKR and RLR subtypes are significantly overexpressed. Based on the ssGSEA scores and univariate Cox analysis, we found that only ADAR and DDR subtypes were significantly associated to the OS of patients in the TCGA-CESC cohort (ADAR: HR = 6.42, 95% CI 1.06–38.71, p = 0.0427; DDR: HR = 3.21, 95% CI 1.05–9.84, p = 0.0409, Fig. 1C). Then, we conducted a tumor immunity analysis and found that the DDR and PACT_TRBP subtypes were consistently associated with a non-inflamed tumor feature. Specifically, we observed a significantly negative correlation between these subtypes and immune scores, CD8+ T cell abundance, as well as expression levels of immune checkpoints (Additional file 1: Fig. S2A–C). On the other hand, RLR and OAS-RNaseL subtypes were associated with a better immunogenic feature, characterized by higher immune scores, greater infiltration of M1 macrophages and CD8 T cells, and increased expression of immune checkpoints (Additional file 1: Fig. S2A–C). Furthermore, we observed no significant difference in the expression levels of ADAR or DDR members among CESC patients with different HPV infection statuses, except for AGO1 (Additional file 1: Fig. S3).

According to ADAR and DDR levels, a consensus clustering algorithm was performed to stratify the tumor samples of cervical cancer with the k = 3 as the number of clusters. Thus, CESC patients in TCGA cohort were dispersed in three distinct clusters named cluster 1, 2, 3 (Fig. 1D). Patients of cluster1 or cluster 2 had worse OS than those from cluster 3 (a median OS: 136.2 months vs 69.8 months vs unreached, p = 0.046, Fig. 1E). PCA further corroborated the classification function of the consensus clustering in the TCGA-CESC cohort (Fig. 1F). In addition, the majority of ADAR- and DDR-related genes exhibited up-regulation in the cluster 1/2. Furthermore, cluster 1/2 had a higher proportion of tumors in advanced stages than cluster 3, including significantly more tumors in T3/T4, N1, M1, stage III+ stage IV, which might explain the poor prognosis (Additional file 1: Fig. S4 and Additional file 2: Table S2).

### Expression patterns of dsRBPs in different clusters

**Fig. 2:**
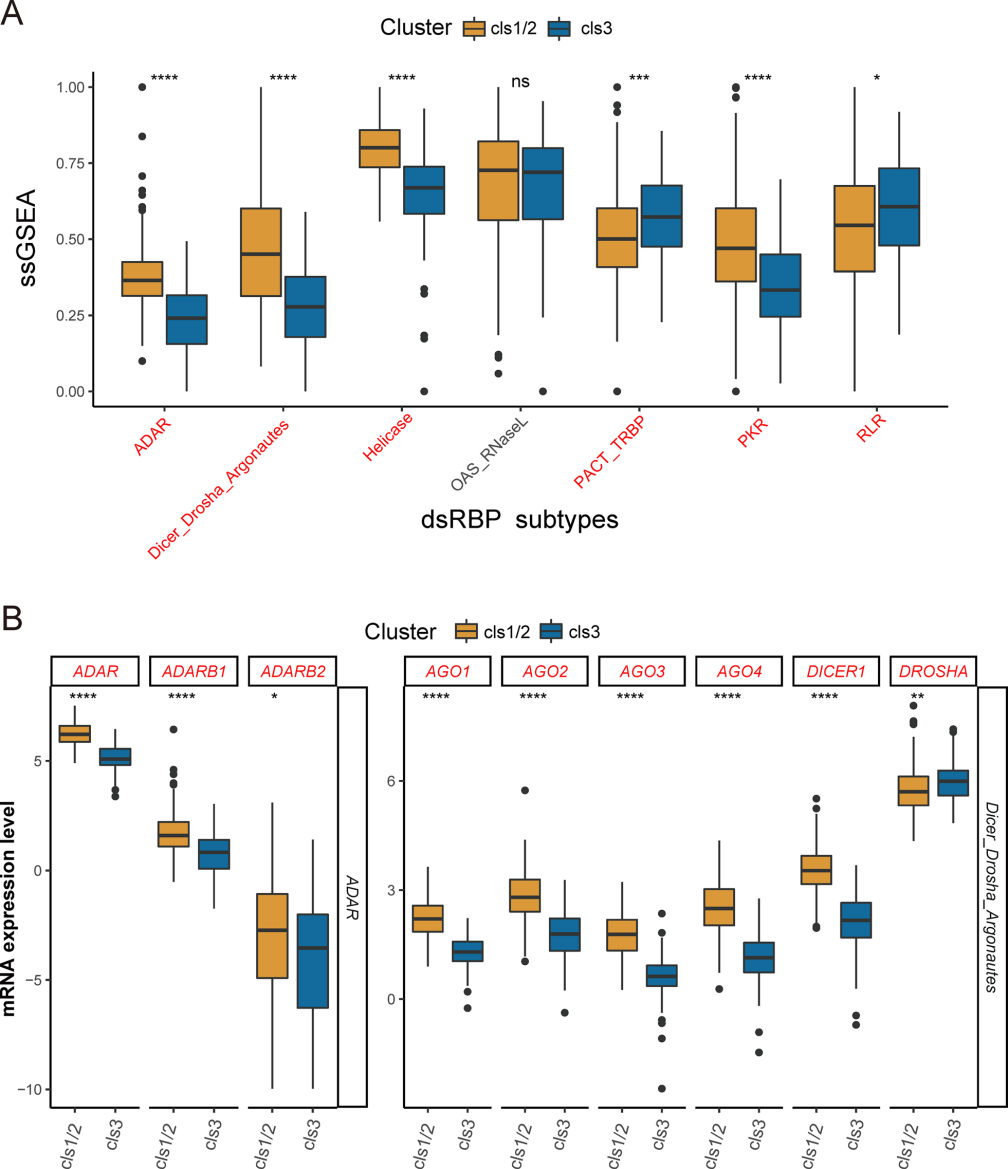
Evaluation of dsRBPs expression in different clusters. **A** Estimation of the ssGSEA scores of seven dsRBPs between cluster 1/2 and 3 (cluster 1/2, n = 234; cluster 3, n = 70). **B** Boxplot showed the different expression levels of ADAR and DDR subfamily members between different clusters. *ssGSEA* single sample Gene Set Enrichment Analysis. *p < 0.05; **p < 0.01; ***p < 0.001; ****p < 0.0001; ns: nonsignificant

Different clusters showed the heterogeneity in the expression profile of dsRBPs. As depicted in Fig. 2A, cluster 1/2 had a higher ssGSEA score in most dsRBPs subtypes, except PACT and RLR (Fig. 2A). Both ADAR (p = 2.4e−15) and DDR (p = 2.8e−12) were notably upregualted in cluster 1/2. Specifically, the expression level of each ADAR and DDR subfamily member was considerably greater in cluster 1/2 (Fig. 2B). In the meantime, there was no significant difference in the distribution of HPV-negative or positive CESC samples between cluster 1/2 and 3 (Additional file 1: Fig. S5).

### dsRBP cluster 3 reveals an enhanced innate immunity

**Fig. 3:**
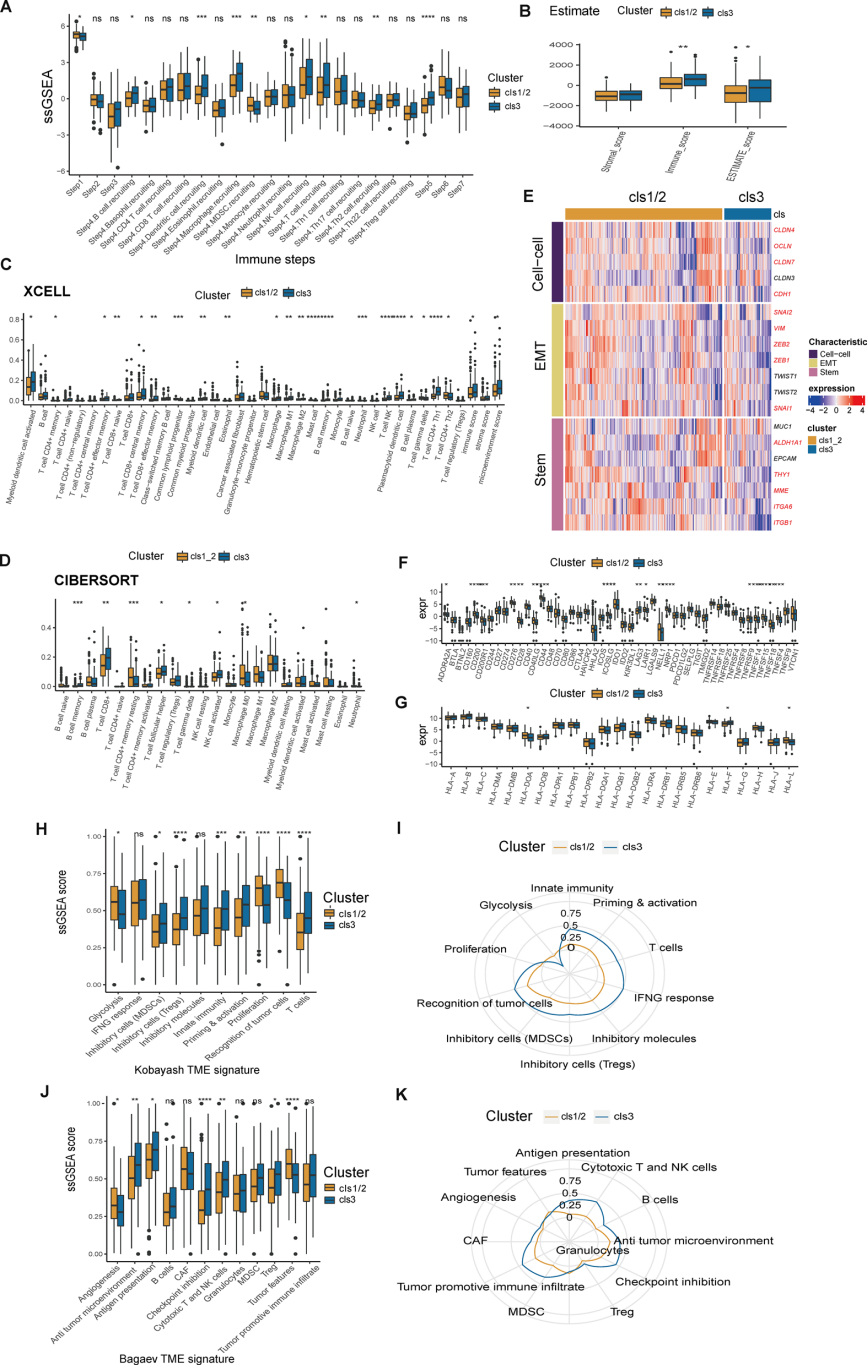
Relationship between dsRBP clusters and TME. **A** Comparison of cancer immune cycle steps between cluster 1/2 and 3 (cluster 1/2, n = 234; cluster 3, n = 70). **B** The stromal score, immune score, and ESTIMATE score of cluster 1/2 and 3. Differences in TIICs enrichment applied by XCELL (**C**) and CIBERSORT (**D**) between distinct clusters. **E** Hub genes involved in the three characteristics were compared across clusters by heat map. The expression levels of 48 immune checkpoints (**F**) and HLA family genes (**G**) between cluster 1/2 and 3. The ssGSEA value (**H**) and immunogram radar plot (**I**) revealed the relationship between distinct clusters and TME signatures generated by Kobayash. The ssGSEA value (**J**) and immunogram radar plot (**K**) revealed the relationship between distinct clusters and TME signatures generated by Bagaev. *TME* tumor microenvironment, *TIICs* tumor infiltrating immune cells, *HLA* human leukocyte antigen. *p < 0.05; **p < 0.01; ***p < 0.001; ****p < 0.0001; ns: nonsignificant

We next assessed differences in the tumor immunology and TME between dsRBPs clusters. First, cluster 3 was presented with increased activity in comprehensive steps involved in the cancer immunity cycle, especially in transport of immune cells to the tumor (step four) and the infiltration of immune cells into the tumor (step five) (Fig. 3A). In line with this result, cluster 3 showed significantly higher immune scores and ESTIMATE scores (Fig. 3B). Using the Xcell (Fig. 3C) and CIBERSOFT, we found a prominent upregulation of tumor-infiltrating immune cells in cluster 3, with significantly increasing levels in memory B cells, CD8+ T cells, NK cells but lower neutrophils levels. When compareing hub genes involved in cell–cell adhesion, endothelial-to-mesenchymal transition (EMT) and stem cell-like feature, we found cluster 3 was characterized by low cell–cell adhesion, EMT and stem cell-like level (Fig. 3E). Then we analyzed the difference in the expression level of immune checkpoints between cluster 1/2 and 3. With only the exception of LAG3, the majority of immune checkpoints, including CD200, CD276, CD28, CD40LG, CD44, ICOSLG, LAIR1, NELL1, NRP1, and TNFSF14, were overexpressed in the cluster 1/2 (Fig. 3F). On the contrary, there was only slight differences in the HLA family genes between different clusters (Fig. 3G). Furthermore, utilizing the immunogram radar plot, we found that cluster 3 was presented with significantly higher activity in comprehensive pathways involved in tumor immunity, except glycolysis (Fig. 3H, I). To support the difference in the immunity, we also investigated the holistic TME feature by using the method described by Bageav et al. Cluster 3 demonstrated increased activity in antigen presentation, checkpoint inhibition, cytoroxic T and NK cells, and Treg, whereas clusters 1/2 shown significantly higher activity in angiogenesis and tumor characteristics (Fig. 3J, K).

### Genomic feature related to dsRBP clusters

**Fig. 4:**
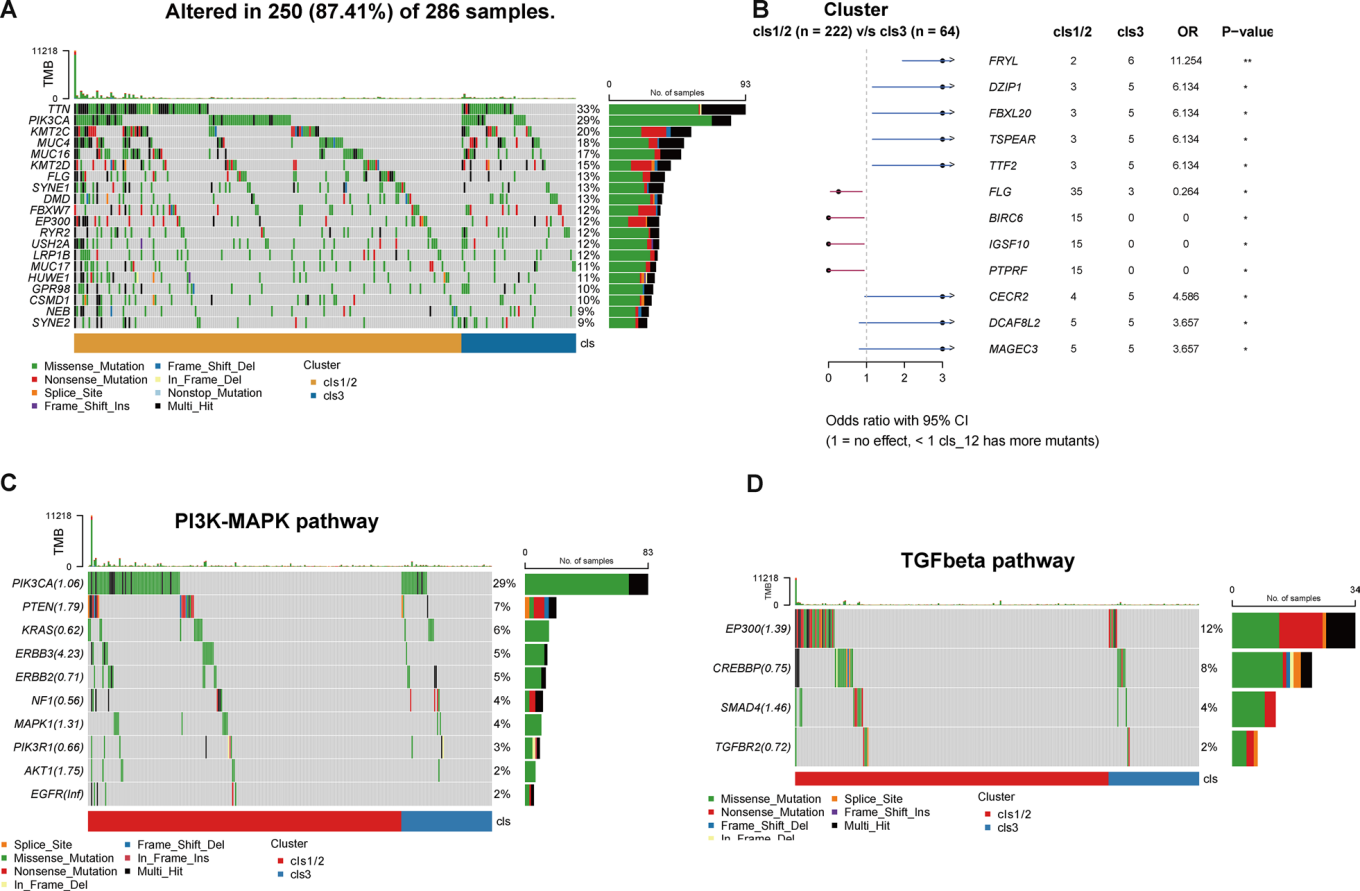
Genomic alterations in cervical cancer associated with dsRBP clusters. **A** Oncoprint plot revealing genomic feature in different dsRBP clusters (cluster 1/2, n = 222; cluster 3, n = 64). Eighteen samples in the TCGA-CESC cohort were excluded due to missing genomic sequencing data. **B** The difference in the prevalent genes between different dsRBP clusters. Genomic alterations within PI3K-MAPK (**C**) and TGFβ (**D**) pathways. *p < 0.05; **p < 0.01

Differences in the prevalence of genomic alterations between cluster 1/2 and 3 were analyzed in the TCGA-CESC dataset, and oncoprint showed *TTN*, *PIK3CA*, and *KMT2C* were the most common in both clusters (Fig. 4A). In addition, as displayed in the forest plot, *FRYL*, *DZIP1*, *FBXL20*, *CECR2*, *DCAF8L2*, *MAGEC3* were significantly more frequently mutated in cluster 3; whereas *FLG*, *BIRC6*, and *IGSF10* alterations were only identified in cluster 1/2 (all p < 0.05, Fig. 4B). As previous studies have shown the prevalent genomic alterations RTK/AKT/MAPK and TGFβ signaling pathways in cervical cancers, then we investigated the genomic difference in these pathways between dsRBP clusters. Notable more genomic alterations involved in RTK/AKT/MAPK signaling pathway were identified in cluster 1/2, especially in *PIK3CA*, *PTEN*, *ERBB3* and *AKT1* (Fig. 4C). Meanwhile, more *EP300* and *SMAD4* alterations were found in cluster 1/2, which were hub genes that participated in the regulation of TGFβ signaling pathway (Fig. 4D).

### Development of a dsRBP signature

**Fig. 5:**
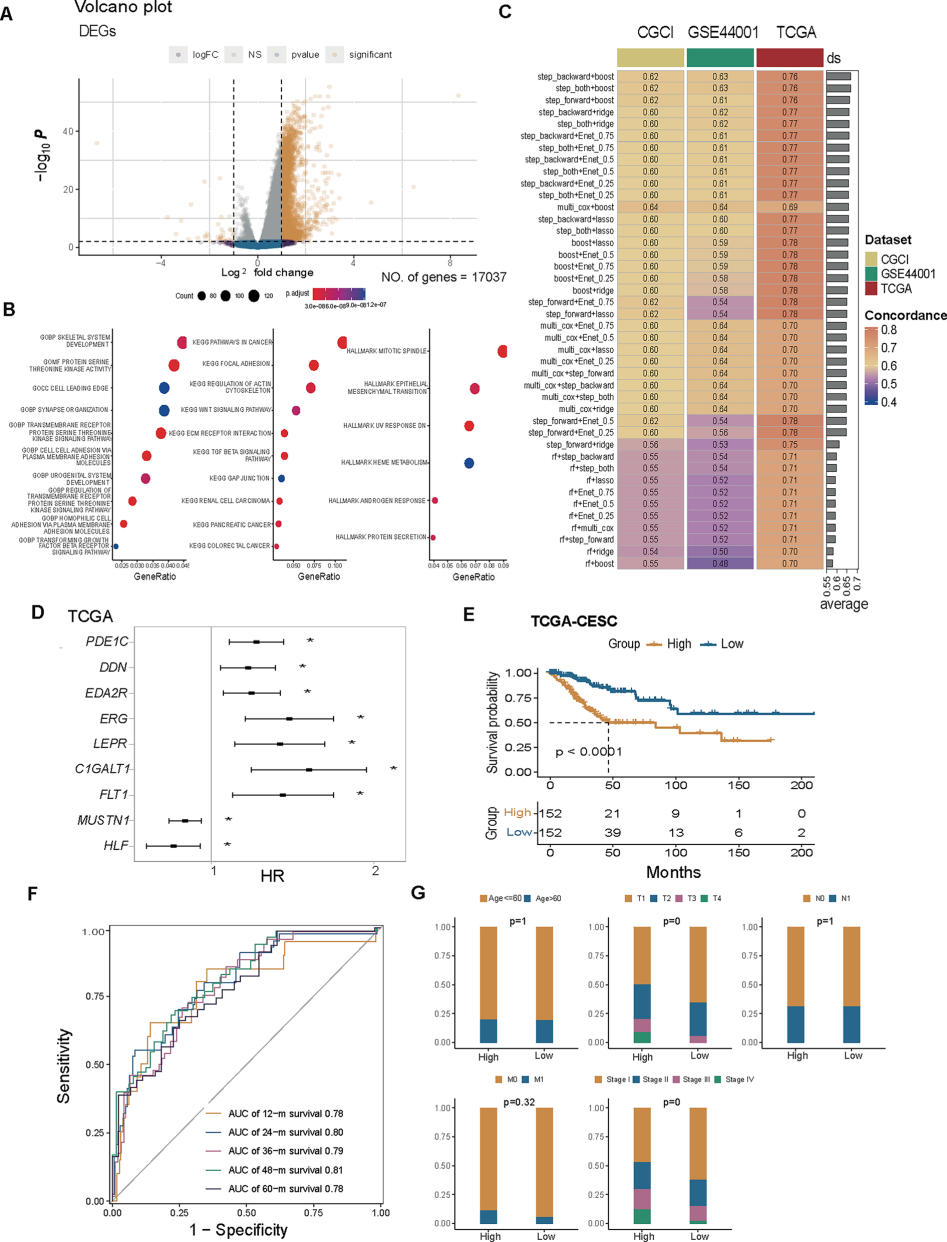
DEGs collection and risk model generation. **A** The volcano plot showed the down-regulated and up-regulated dsRBP cluster-associated DEGs. **B** Functional enrichment analysis of DEGs. **C** There were 42 combinations of machine learning algorithms for the risk model in CGCI-HTMCP-CC, GSE44001, and TCGA-CESC datasets. **D** The association between signature genes and the OS of cervical cancer in the TCGA-CESC cohort (high-risk group: n = 152; low-risk group, n = 152). **E** Kaplan–Meier curve of cervical cancer patients in TCGA-CESC dataset. **F** ROC curve and AUC of 12-, 24-, 36-, 48- and 60-month survival in TCGA-CESC cohort. **G** The relationship between clinic variables and two groups, including age, T stage, N stage, M stage, and neoplasm disease stage. *DEGs* differentially expressed genes, *ROC* receiver operating characteristic, *AUC* area under the curve. *p < 0.05

To develop a prognostic dsRBP signature, we analyzed dsRBP cluster-associated DEGs, of which 39 were down-regulated and 2931 were up-regulated (Fig. 5A and Additional file 2: Table S3). Consistent with previous dsRBP clusters-related feature, these DEGs were mainly enriched in cell adhesion and microenvironment [focal adhesion, extracellular matrix (ECM), gap junction], WNT, TGFβ and EMT pathways (Fig. 5B). Then, we obtained the prognostic cluster-associated DEGs based on univariate Cox analysis (Additional file 2: Table S4), which were further filtered by comparing the expression levels of tumor and normal tissues (Additional file 2: Table S5). Additionally, we performed 6 machine learning algorithms, including stepwiseCox, CoxBoost, Enet, LASSO, Ridge, random forest, combined to construct a risk model depending on optimal DEGs, which can identify the most robust and stable risk model with the highest C-index in the TCGA-CESC cohort, GSE44001 cohort and CGCI-HTMCP-CC cohort (Fig. 5C and Additional file 2: Table S6). Finally, a risk model with the best performance was built based on stepwise Cox and CoxBoost, in which stepwise Cox identified 9 most important DEGs (PDE1C, EDA2R, DDN, LEPR, C1GALT1, MUSTN1, ERG, HLF, and FLT1), and the CoxBoost screened out the most robust risk model. The majority of these signature genes (7/9) were associated with a worse survival with the exception of MUSTN1 and HLF (Fig. 5D). The formula of the risk model was as follows:

$$\begin{aligned} {\text{dsRBP}}\;{\text{risk}}\;{\text{core}} & = {\text{Expression}}\;{\text{of}}\;{\text{PDE}}1{\text{C}} \times \left( {0.8063947} \right) + {\text{Expression}}\;{\text{of}}\;{\text{EDA}}2{\text{R}} \times \left( {0.4478117} \right) + {\text{Expression}}\;{\text{of}}\;{\text{DDN}} \times \left( {0.6091578} \right) \\ & \quad + {\text{Expression}}\;{\text{of}}\;{\text{LEPR}} \times \left( {0.3721565} \right) + {\text{Expression}}\;{\text{of}}\;{\text{C}}1{\text{GALT}}1 \times \left( {0.3232040} \right) + {\text{Expression}}\;{\text{of}}\;{\text{MUSTN}}1 \times \left( { - 0.6317096} \right) \\ & \quad + {\text{Expression}}\;{\text{of}}\;{\text{ERG}} \times \left( {0.3797116} \right) + {\text{Expression}}\;{\text{of}}\;{\text{HLF}} \times \left( { - 0.9292308} \right) + {\text{Expression}}\;{\text{of}}\;{\text{FLT}}1 \times \left( { - 0.1869477} \right). \\ \end{aligned}$$

Then, patients in the TCGA-CESC cohort were dichotomized into high- and low-risk groups based on median risk score (Additional file 2: Table S7). The Kaplan–Meier curve confirmed that the OS of the high-risk group was significantly shorter than that of the low-risk group in the TCGA-CESC dataset (median OS: 46.47 vs unreached, p < 0.0001, Fig. 5E). In the meantime, the 12-, 24-, 36-, 48-, and 60-month prediction accuracy was 0.78, 0.80, 0.79, 0.81, and 0.78, respectively (Fig. 5F). Meanwhile, the stratification survival analysis in the TCGA-CESC cohort demonstrated that the patients in the high-risk group had significantly worse OS for all clinical parameters (Additional file 1: Fig. S6), and the clinical feature information of the high- and low-risk groups was displayed in Additional file 2: Table S8. Univariate and multivariate Cox regression analysis further revealed that dsRBP risk score was the only independent risk factor (Additional file 1: Fig. S7). High-risk group was presented with a higher proportion of patients with advanced tumor stage and neoplasm disease stage (Fig. 5G).

### Validation of the dsRBP signature

**Fig. 6:**
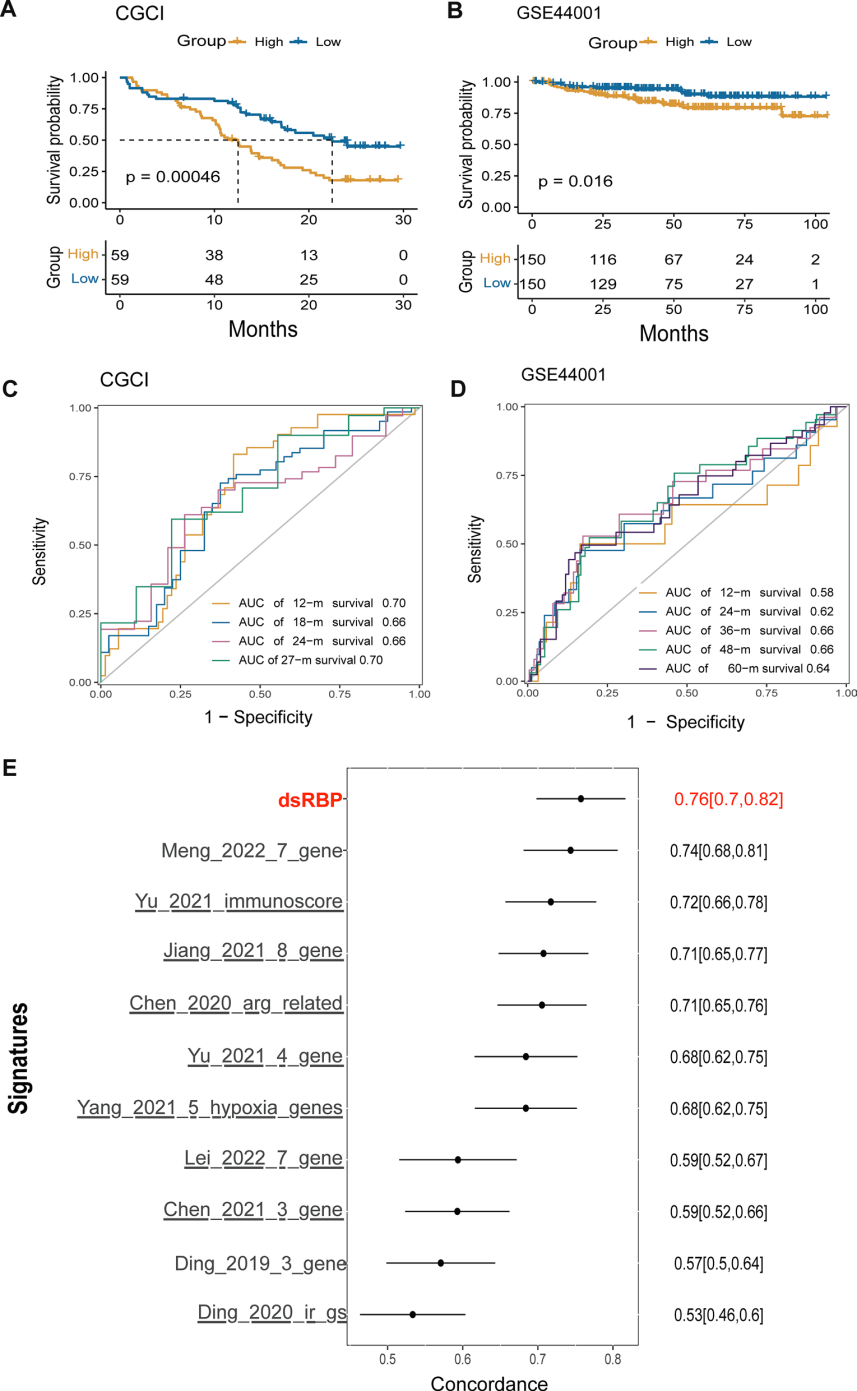
The validation of the risk model for predicting the prognosis of cervical cancer. Survival analysis showing the OS of cervical cancer patients in the CGCI-HTMCP-CC dataset (high-risk group: n = 59; low-risk group, n = 59, **A**) and the DFS of cervical cancer patients in the GSE44001 dataset (high-risk group: n = 150; low-risk group, n = 150, **B**). **C** The AUCs for 12-, 18-, 24-, and 27-month ROC in the CGCI-HTMCP-CC cohort. **D** The AUCs for 12-, 24-, 36-, 48- and 60-month ROC in the GSE44001 cohort. **E** Comparison of the predictive ability of the dsRBP signatures with 10 previously reported risk models for cervical cancer. *DFS* disease-free survival

GSE44001 and CGCI-HTMCP-CC datasets served as two external validation datasets to evaluate the prognosis prediction capacity of the dsRBP signature (Additional file 2: Tables S9, S10). The survival analysis showed that the high-risk group had a worse OS in CGCI-HTMCP-CC cohort (a median OS: 12.50 vs 22.47 months, p = 0.00046; Fig. 6A), and a significantly worse disease-free survival (DFS) in the GSE44001 cohort (a median DFS: unreached vs unreached months, p = 0.016; Fig. 6B). Besides, the AUC for prediction OS at 12-, 18-, 24-, and 27-month were 0.70, 0.66, 0.66, and 0.70, respectively, in CGCI-HTMCP-CC dataset (Figs. 6C). The AUC for predicting DFS at 12-, 24-, 36, 48, and 60 month were 0.58, 0.62, 0.66, 0.66, and 0.64, respectively, in GSE44001 dataset (Fig. 6D). In addition, we evaluated the predictive capacity of the dsRBP signature to that of 10 previously reported risk evaluation models in cervical cancer, and found that our established dsRBP signature outperformed those risk models in predicting survival (Fig. 6E).

### Expression feature of the signature-related genes and dsRBP subtypes

**Fig. 7:**
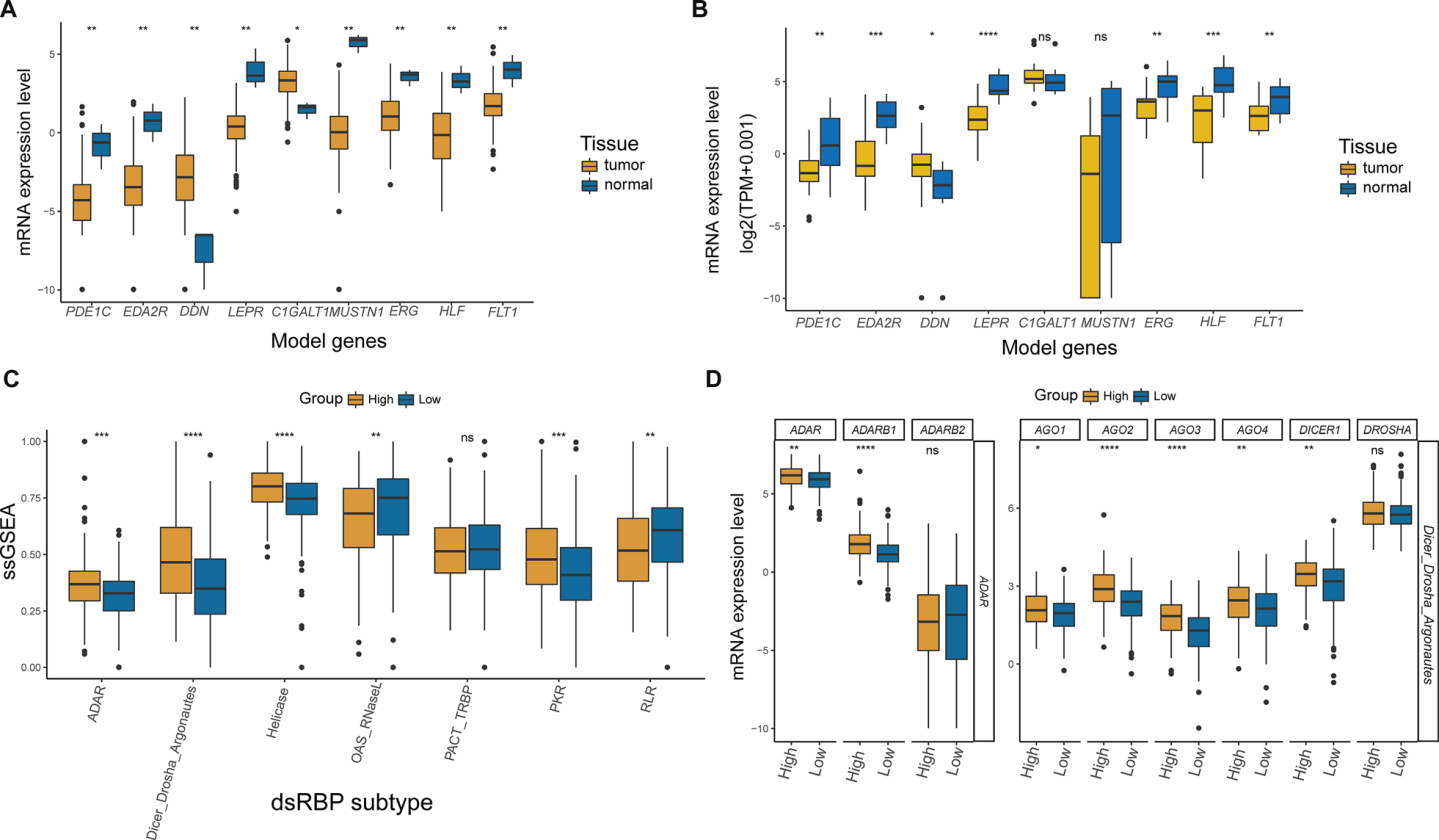
Analysis of the expression profiles of risk model-related genes and dsRBP subtypes. Nine signature-related genes were differentially expressed between cancer and normal tissues in TCGA-CESC (normal, n = 3; cancer, n = 307; **A**) and local FUSCC cohort (normal, n = 15; cancer, n = 15; B). **C** The ssGSEA scores of dsRBPs subtypes between high- and low-risk groups. **D** The expression level of ADAR-related and DDR-related genes between two groups (high-risk group: n = 152; low-risk group, n = 152). *p < 0.05; **p < 0.01; ***p < 0.001; ****p < 0.0001; ns: nonsignificant

Subsequently, we evaluated the expression feature of the nine signature-related genes in cervical tumor tissues and normal tissues. Intriguingly, nearly all signature-related genes were significantly downregulated in the tumor tissue in both the TCGA and local cohort, except DDN and C1GALT1 (Fig. 7A, B). The high-risk group showed distinguishing characteristics of dsRBP subtypes that corresponded to those of dsRBP clusters 1/2, such that high-risk group had significantly higher ADAR, DDR, Helicase, OAS RNasel, and PKR ssGSEA scores, whereas low-risk group had significantly higher RLR ssGSEA scores (Fig. 7C). Most ADAR-related and DDR-related genes exhibited significantly elevated expression level in the high-risk group, including ADAR, ADARB1, AGO1, AGO2, AGO3, AGO4, and DICER1 (Fig. 7D). When comparing HPV-positive and HPV-negative samples, HPV-negative samples had significantly higher dsRBPs risk scores (distribution of HPV-negative or positive CESC samples between cluster 1/2 and 3 (Additional file 1: Fig. S8A). Similarly, when comparing different hierarchical HPV calls, the trend in difference was consistent, but without statistical significance (Additional file 1: Fig. S8B). Additionally, hierarchical HPV clade-negative patients also had higher dsRBPs risk scores compared to those with A7 (n = 120) and A9 (n = 45) (Additional file 1: Fig. S8C). Furthermore, we did not observe any significant differences between CESC patients with (n = 141) or without HPV DNA integration (Additional file 1: Fig. S8D).

### Profiling the cell subpopulation associated with dsRBP signature

**Fig. 8:**
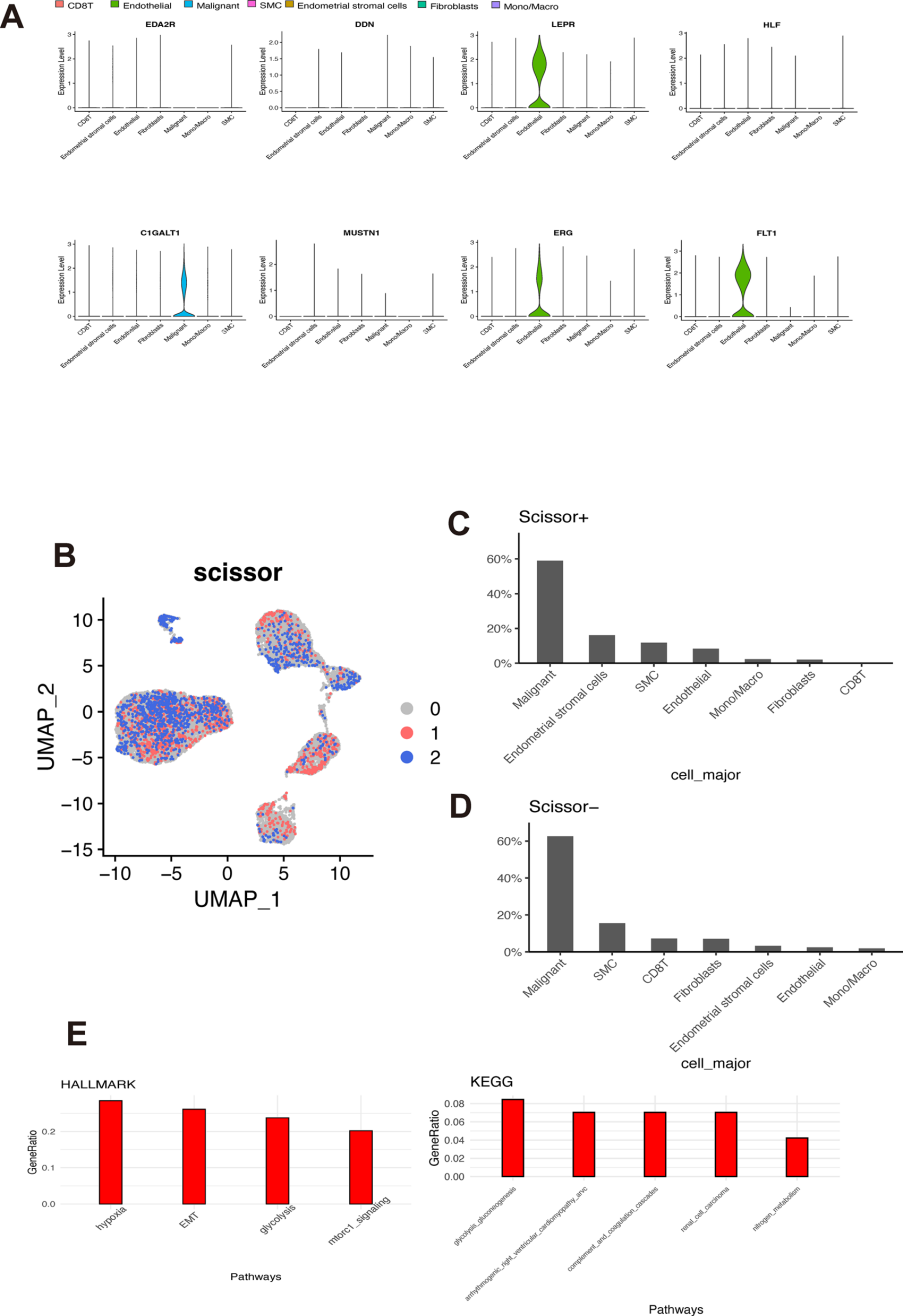
Profiling the cell subpopulation associated with dsRBP signature. **A** Difference in the expression level of hub genes in different cell types from GSE168652 dataset. **B** UMAP visualization of the Scissor-selected cells. The red and blue dots are Scissor+ and Scissor− cells, which were associated with the dsRBP signature or not, respectively. Distribution of Scissor+ (**C**) and Scissor− cells (**D**) among various cell types. **E** pathway enrichment of differentially expressed genes between Scissor+ and Scissor− cells by HALLMARK and KEGG analysis

Among the identified hub genes, we found that only C1GALT1 was significantly overexpressed in the tumor cells, whereas LEP4, ERG, and FLT1 were primarily expressed in the endothelial cells in scRNA-seq dataset (Fig. 8A). To evaluate the association between the bulk RNA sequencing-derived dsRBP risk signature and scRNA-seq profile, we used the recently published computational method Scissor. This analysis identified 1202 Scissor+ cells and 1222 Scissor− cells in the GSE168652 dataset (Fig. 8B). While the majority of Scissor+ and Scissor− cells were malignant cells, the other Scissor+ cells were enriched in endometrial stromal cells, SMCs, and endothelial cells (Fig. 8C), while the other Scissor− cells showed enrichment for SMCs, CD8+ T cells, and fibroblasts (Fig. 8D). Furthermore, a total of 125 genes were differentially expressed between Scissor+ and Scissor− malignant cells, mainly enriched in hypoxia, epithelial-mesenchymal transition, glycolysis, and mTORC1 signaling pathways (Fig. 8E).

### TME and tumor immunity related to dsRBP signature

**Fig. 9:**
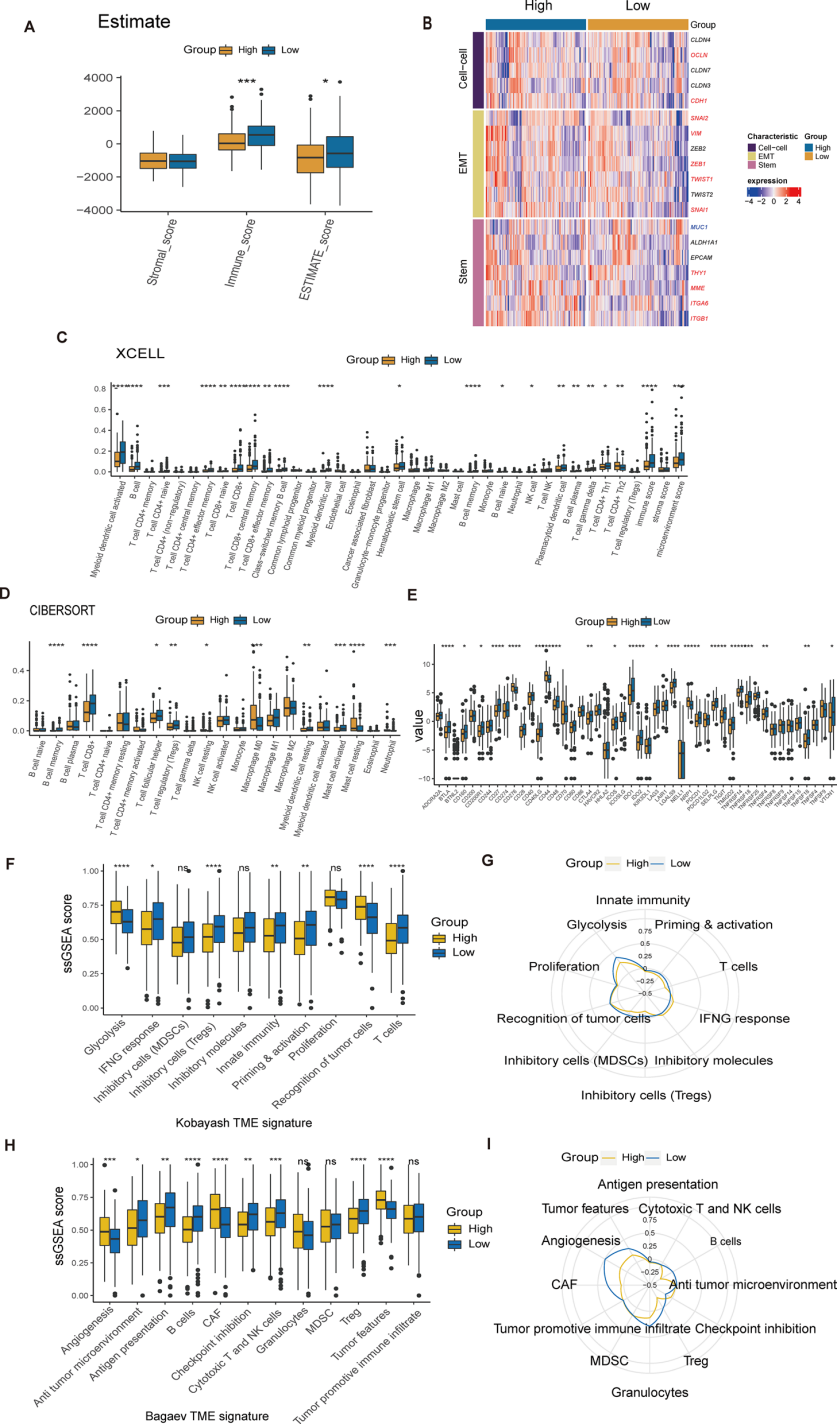
The evaluation of high- and low-risk groups in TME. **A** The stromal score, immune score, and ESTIMATE score of the two groups (high-risk group: n = 152; low-risk group, n = 152). **B** The heat map exhibited the difference between high- and low-risk groups in the hub genes involved in the three features. Differences in TIICs enrichment performed by two algorithms, including XCELL (**C**) and CIBERSORT (**D**). **E** The expression levels of immune checkpoints between high- and low-risk groups. The ssGSEA score (**F**) and immunogram radar plot (**G**) displayed the association between two groups and TME signatures constructed by Kobayash. The ssGSEA score (**H**) and immunogram radar plot (**I**) displayed the association between two groups and TME signatures constructed by Bagaev. *p < 0.05; **p < 0.01; ***p < 0.001; ****p < 0.0001; ns: nonsignificant

Similar to the feature of dsRBP cluster 1/2, high-risk group had a significantly lower immune score and ESTIMATE score than the low-risk group (Fig. 9A). In the meanwhile, high-risk presented higher expression levels of genes involved in cell–cell adhesion (OCLN, CHD1), EMT (SNAI1, SNAI2, VIM, ZEB1, TWIST1) and stemness (THY1, MME, ITGA6 and ITGB1, Fig. 9B). When we evaluated the difference in TIIC abundance between high- and low-risk groups, we discovered that the high-risk group was associated with a relative “cold” TME. In combination with the xCELL (Fig. 9C) and CIBERSOFT (Fig. 9D) results, the high-risk group had a significantly decreased abundance of CD8+ T cells, memory B cells, and NK cells. Regarding the expression level of immune checkpoints, the high-risk group had significantly higher levels of CD276, CD44, and NRP1, whereas the low-risk group had significantly higher levels of a greater number of immune checkpoints, such as PDCD1, CTLA4, IDO1, IDO2, LAG3, and ICOS (Fig. 9E). In addition, high-risk group had significantly elevated levels in glycolysis and recognition of tumor cells, but lower levels in IFNg response, inhibitor cells (Tregs), innate immunity, priming and activation of immunology and T cells (Fig. 9F, G). In accordance with our previous analyses, Bageav analysis also showed high-risk group had significantly higher levels in angiogenesis, cancer associated fibroblasts (CAFs) and tumor feature but lower levels in anti-tumor microenvironment, antigen presentation, B cells, checkpoint inhibition, cytotoxic T and NK cells and Tregs (Fig. 9H, I).

### Somatic mutation and CNVs related ot risk model

**Fig. 10:**
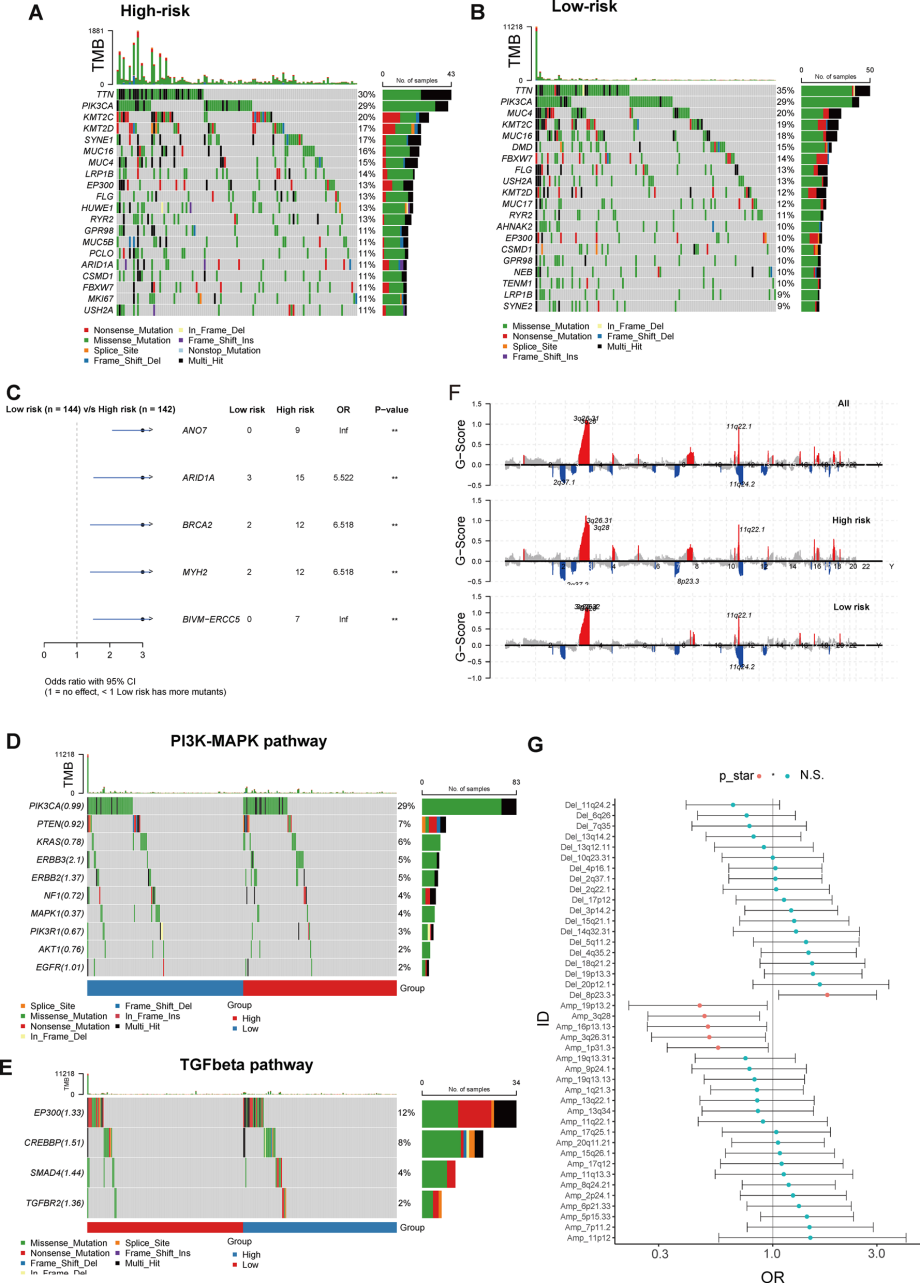
Identification of genetic mutations underlying the risk model. The waterfall plots of the high-risk group (n = 144, **A**) and low-risk group (n = 142, **B**) in the TCGA cohort. **C** Top 5 frequently mutated genes in high- and low-risk groups. Gene mutations in the PI3K-MAPK (**D**) and TGFβ (**E**) pathways. **F** Focal peaks showed CNV types: red (amplification) and blue (deletion). **G** The incidence of amplification or deletion of genomic regions in the high- and low-risk groups. *CNV* copy number variation. *p < 0.05; **p < 0.01

We have conducted an evaluation of the mutated prevalence in CESC samples from the TCGA database and have found that the hub genes are seldom altered in this cancer type. Specifically, we have only identified genomic alterations in LEPR, FLT1, PDE1C, and ERG, with a prevalence rate beyond 1% (Additional file 1: Fig. S9). And in 25 cervical cancer samples in local cohort who preformed whole exon sequencing, there was no somatic alteration in those hub genes identified. In TCGA-CESC cohorts, the most prevalent genes were similar between high- and low-risk groups, showing *TTN*, *PIK3CA*, and *KMT2C* as the most prevalent alterations (Fig. 10A, B). Compared to the low-risk group, high-risk group had significantly higher prevalence in *ANO7*, *ARID1A*, *BRCA2*, *MYH2*, and *ERCC5* (Fig. 10C). Among PI3K–MAPK pathway genes, the high-risk group had a higher prevalence of *ERBB2* and *ERBB3*, but a lower frequency of *MAPK1*, *PIK3R1*, and *AKT1* alterations (Fig. 10D). In contrast, the high-risk group had a higher frequency of genes involved in the TGFβ signaling pathway (Fig. 10E). Subsequently, we analysed the difference in the copy number variants between high- and low-risk groups (Fig. 10F). There were no distinguishing characteristics between the high- and low-risk groups; however, the high-risk group had significantly more Amp at 19p13.2, 3q28, 1p31.3, and 19p13.31 and less Del at 8p23.3 (Fig. 10G).

### Estimation of immunotherapy and chemotherapy response associated with dsRBP signature

**Fig. 11:**
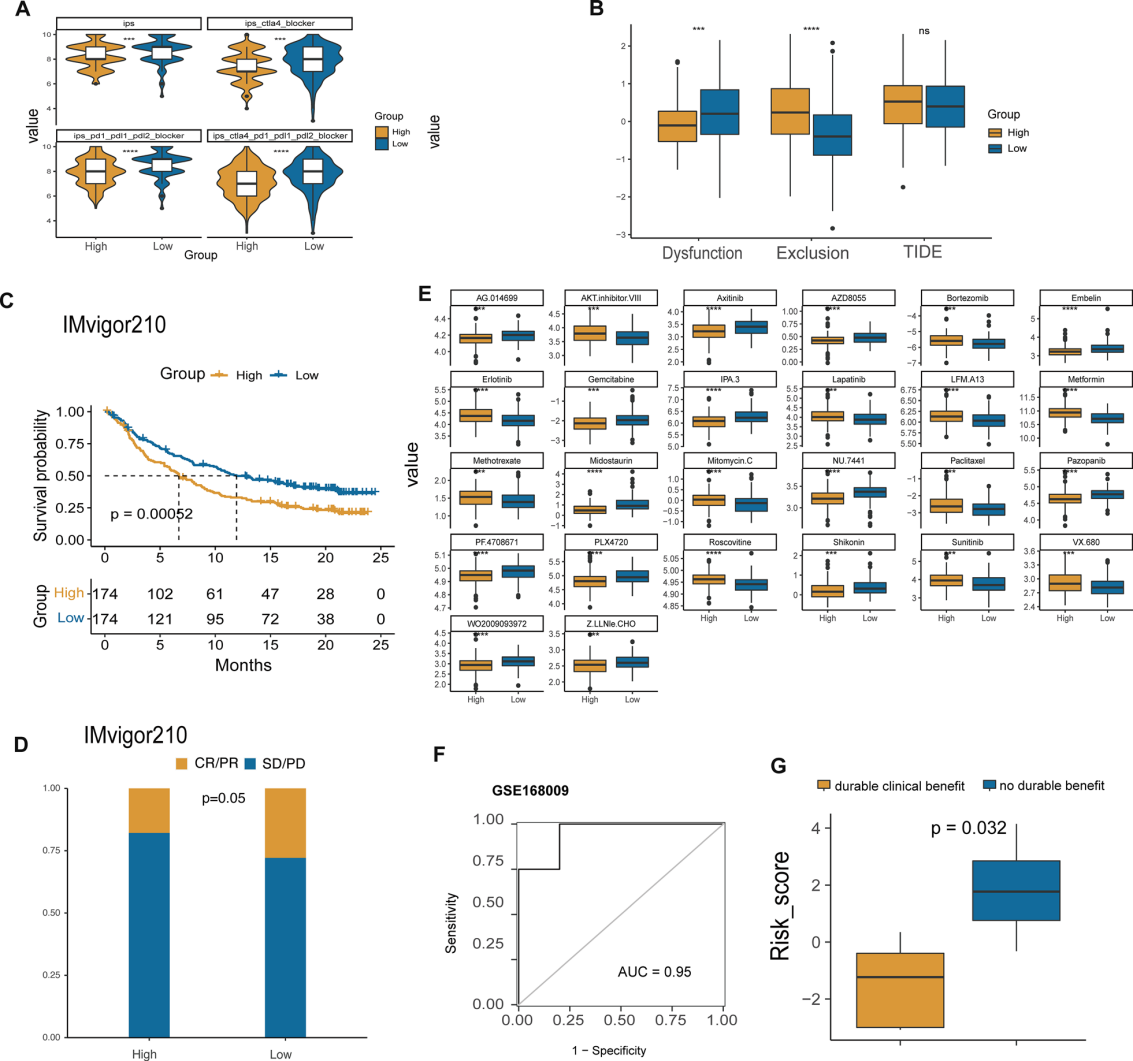
Predictive capacity of the signature in immunotherapy response and chemosensitivity. **A** IPS difference between high- and low-group with different statuses of CTLA-4, PD-1, PD-L1, and PD-L2 (high-risk group: n = 152; low-risk group, n = 152). **B** The difference in T cells dysfunction score, T cells exclusion score, and TIDE score between the two groups. **C** Difference in the overall survival between high- and low-risk groups in the IMvigor210 dataset. **D** Difference in the objective response rate between high- and low-risk group in the IMvigor210 dataset (high-risk group: n = 174; low-risk group, n = 174). **E** Chemotherapy drugs in cervical cancer with distinct IC50 values between the two groups. **F** AUC value in GSE168009 cohort. **G** Response to CCRT based on risk score in GSE168009 cohort (durable clinical benefit, n = 5; no durable benefit, n = 4). *IPS* immunophenoscore, *TIDE* tumor immune dysfunction and exclusion, *IC50* half-maximum inhibitory concentration, *CCRT* concurrent chemoradiotherapy, *CR* complete response, *PR* partial response, *SD* stable disease, *PD* progression dsiease. ***p < 0.001; ****p < 0.0001; ns: nonsignificant

According to Fig. 11A, the IPS score, IPS–PD1/PDL1/PDL2 blocker score, IPS-CTLA4 blocker score, and IPS-CTLA4 and PD1/PDL1/PDL2 blocker score were all significantly higher in the low-risk group, indicating that CESC patients with lower risk score might respond better to immunotherapy. Meanwhile, the high-risk group had a lower T cell dysfunction score, but a significantly higher T cell exclusion score (Fig. 11B). Based on the IMvigor210 cohort, the patients with high-risk score had a significantly worse OS than those of low-risk score (p = 0.00052, Fig. 11C). In the meantime, low-risk group had significantly higher proportion of objective responders than the high-risk group (Fig. 10D). Then, we analyzed the sensitivity to 26 chemotherapeutic drugs based on the stratification of risk score, and the results revealed that high-risk group was more sensitive to gemcitabine, axitinib, pazopanib and AZD8055; while low-risk group had higher sensitivity to pacitaxel, erlotinib, lapatinib and sunitinib (Fig. 11E). On the other hand, the risk score had good predictive ability in the cervical cancer patients treated with concurrent chemoradiotherapy (CCRT) with an AUC value of 0.95 in the GSE168009 dataset (Fig. 11F). Accordingly, the cervical patients with durable clinical benefits treated with CCRT exhibited significantly lower risk scores (Fig. 11G).

## Discussion

dsRNA typically accumulates as a result of viral infection, which can activate the innate immune system to distinguish it. Despite significant progress in understanding how dsRNA and dsRBP function, their role in cervical cancer remains unclear. While previous studies have extensively demonstrated the positive relationship between dsRBP and viral infection, it is important to note that dsRBP also plays crucial roles in uninfected cells for maintaining normal biological processes. Therefore, it remains unknown whether there are differences in the expression features of different dsRBP subtypes between CESC cases with or without HPV infection. In our study, we observed distinct expression profiles in dsRBP subtypes associated with HPV infection, particularly in DDR, PACT_TRBP, OAS_RNAseL, and RLR. Previous research indicated that HPV-positive cervical carcinoma cell lines exhibit higher levels of DROSHA and DICER mRNA compared to HPV-negative cervical carcinoma cell lines, suggesting dysregulation of DDR-related miRNA levels [35]. However, in our current study, we found a significant downregulation of DDR levels in the HPV-positive CESC samples from the TCGA-CESC cohort. Additionally, no significant difference in the mRNA expression levels of DROSHA and DICER was observed between HPV-positive and HPV-negative samples. The discrepancy in DDR ssGSEA score primarily stemmed from differences in the mRNA expression level of AGO1, which exhibited significantly elevated expression in the HPV-negative CESC samples. Furthermore, Wang and her colleagues also discovered a significant association of OAS3 with CIN3/cancer, including viral infections in CESC. This finding supports the increased expression level of OAS_RNaseL in HPV-positive samples [36]. RNASEL, an enzyme central to interferon-related antiviral and apoptotic responses, has been linked to increased risk for CESC, head and neck squamous cell carcinomas, and breast cancer through single nucleotide polymorphisms such as rs3738579 [37]. RIG-I plays a crucial role as an immune sensor in inducing intrinsic apoptotic cell death and cisplatin-mediated cell killing in CESC. This activation is associated with the activation of natural killer cells in the tumor microenvironment [38]. However, HPV E6 has been found to inhibit the induction of IFN mediated by RIG-I. This inhibition is primarily achieved through targeting the upstream factors TRIM25 and USP15 [39]. Therefore, the observed increase in the expression level of RLR subtype, particularly RIG-I and MDA5, in HPV-positive CESC samples may be attributed to the IFN-stimulated antiviral process. Interestingly, we discovered that HPV-negative CESC patients exhibited significantly higher dsRBPs risk scores compared to HPV-positive patients. This finding aligns with the observation that HPV-negative CESC patients generally have a poorer prognosis [40].

In our research, we observed a significant downregulation of ADAR in cervical tumor tissues, while Helicase, OAS_RNAsel, PKR, and RLR were upregulated. This finding aligns with the regulation network of dsRNA sensors and modulators in innate immunity, where ADAR mainly functions as a counterpart to these subfamilies [41, 42]. Notably, ADAR and DDR were the only two dsRBP subtypes significantly associated with the clinical outcomes of CESC. ADAR is responsible for the modification of adenosines to inosines in dsRNAs, and there are three ADAR proteins (ADAR1-3) in humans [43]. Previous studies have also supported the role of ADARs as novel oncogenes in CESC, associated with poor prognosis, unfavored pathological factors, and angiogenesis [44, 45]. DDR comprises three conserved RNases involved in RNA interference regulation, playing a role in miRNA and siRNA biogenesis and related mRNA silencing [46, 47]. Dicer, a member of the DDR family, has been identified as a risk factor in CESC, significantly associated with distant metastasis and disease recurrence [48]. Additionally, Drosha exhibits upregulated expression due to copy number gain at chromosome 5, promoting tumor progression in CESC [49]. Furthermore, AGO3 has been found to enhance the proliferation and growth of CESC cells through the Wnt/β-catenin signaling pathway [50]. These studies support our findings regarding the association of ADAR and DDR with cervical cancer prognosis. Interestingly, both ADAR and DDR showed relatively downregulated expression in tumor tissues compared to normal cervical tissues in both TCGA and FUSCC cohorts, consistent with previous study results [51].

Based on the pattern of ADAR and DDR expression, we classified cervical cancer patients into three clusters, with cluster 3 showing the best survival, confirming the predictive function of ADAR and DDR. Cluster 3 had significantly lower expression level of ADAR and DDR but more TIICs presented in the tumor microenvironment. In the last decades, TIICs are being recognized as play an essential role in TME and are involved in the development of many types of tumors, hence investigation the value of immune cell infiltrates in TME is crucial to provide novel therapeutic methods and improving immunotherapy response rates for cancer [52]. Previous studies have identified the pivotal role of TIICs in the regulaiton of disease development of cervical cancer [53, 54]. Both CIBERSOFT and Xcell revealed that cluster 3 had significantly higher level of CD8+ T cells, which is not only a prognostican factor [55] but also associated with the response to ICIs in cervical cancer [56]. On the other hand, recent research has suggested that neutrophils, as the key regulator of cancer, can influence the inflammation in cancer and induce the angiogenesis of cancer, thereby accelerating tumor initiation, proliferation, growth, and metastasis [57, 58]. Besides, cancer associated fibroblast is identified as a microenvironmental cell and offers metabolic support for a malignant tumor, which has the biological functions of immunogenic and immunosuppressive [59], and endothelial cells are proven to be an emerging important factor that participates in the regulation of cancer development [60]. In the present research, the abundance of neutrophils, cancer associated fibroblast, and endothelial cells were elevated in cluster 1/2, which might explain the poor outcomes of patients in cluster 1/2.

Previous research reveals that HPV infection, a crucial step in the initiation and development of cervical cancer will generate an immunosuppressive microenvironment and negate host antitumor immunity; therefore, immunotherapy is a promising developing treatment option for individuals with CESC [61, 62]. However, even anti-PD-1/PD-L1 treatments for CESC have been approved by FDA, the majority of CESC patients have limited antitumor efficacy to the monotherapy of these regimes [63]. Then, combination with other therapies, particularly those targeted at other immune checkpoints to elude immune surveillance in cervical cancer, has garnered significant interest and been the subject of extensive preclinical and clinical investigations [64]. In comparison to cluster 3, we discovered that the more immune checkpoints, such as ADORA2A, CD160, CD200R1, CD276, CD28, CD40LG, CD44, ICOSLG, NELL1, NRP1, TNFSF14, TNFSF18, and TNFSF4, were more abundant in cluster 1/2. Recent work demonstrated that CD28 contributes to the development of cervical cancer and can serve as a prognostic marker for cervical cancer [65]. Furthermore, CD44, as the specific cell adhesion molecule, is proven to increase the migration and invasion of cervical cancer [66]. It may suggest that therapy targeted at these overexpressed immune checkpoints may remold the immunosuppressive microenvironment and then enhance the effect of anti-PD-1/L1 therapy in patients of cluster 1/2.

In the meantime, genomic mutations participate in the growth and survival of tumor cells by giving the tumor a selective advantage and providing valuable insights for early cancer diagnosis, disease monitoring and treatment [67]. In cluster 1/2, the incidence of *FLG*, *BIRC6*, and *IGSF10* mutations were significantly higher, and it is reported that *FLG* contributes to the biological activity of barrier function and associates with the poor prognosis of cervical cancer [68]. Furthermore, when focused on the TGF-beta pathway, we found a significant enrichement of alterations in *EP300* and *SMAD4* in the cluster1/2. *SMAD4* is a known tumor suppressor in cervical cancer and an essential regulator of the TGF-beta pathway, which has been widely linked to the progression of disease [69, 70].

To further apply the prognostic potential of dsRBPs, we constructed a risk model for CESC patients. Through stepwise Cox and CoxBoost analyses, we developed a risk model using the expression levels of nine cluster-related DEGs. The predictive ability of the dsRBP-based risk model was robust, as demonstrated in both the training and validation cohorts, which outperformed ten previously published risk models for CESC. Among the genes included in our risk model, PDE1C, EDA2R, LEPR, C1GALT1, MUSTN1, and HLF were identified for the first time to be associated with the prognosis of CESC, warranting further investigation. Previous studies have linked several of these genes to malignant progression in various cancers, including cervical cancer. For instance, DDN has been found to be overexpressed in CESC tissues, and knockdown of the lncRNA DDN-AS1 has been shown to inhibit tumor proliferation and migration [71]. ERG, a regulator of the glycolysis process, has been implicated in enhancing the growth and invasion of CESC through its impact on aerobic glycolysis capacity [72]. Furthermore, FLT1 has been identified as a prognostic biomarker for CESC [73]. C1GALT1 has been associated with the development of gastric carcinogenesis, with overexpression shown to stimulate the PI3K/AKT pathway and regulate integrin α5O glycosylation [74]. HLF has been reported to play a role in regulating the development of triple-negative breast cancer by activating tumor cell macrophage crosstalk, which also affects chemotherapeutic resistance [75]. These findings highlight the potential clinical relevance and significance of our dsRBP-based risk model for cervical cancer patients.

We also investigated the application of the established signature in selecting therapeutic regimens. Studies also have shown that TIDE and or IPS is a highly effective predictor for treatment response to anti-PD-1 and CTLA-4 therapies [32]. The low-risk group displayed lower T cells exclusion score, demonstrating the low-risk group might benefit more from immunotherapy. The evevalted IPS score in the low-risk group further supports the notion that this group exhibits a more favorable response to immunotherapy. These and the findings from IMVigor210 dataset all suggests that our risk model could serve as a potential therapeutic target for estimating the immunotherapy response in patients with CESC. Besides, CCRT is not only the optimal treatment method but also the standard care for locally advanced CESC patients, which can improve the survival rate [76, 77]. Our study investigated the relationship between CCRT and the risk model in the GSE168009 dataset and indicated that the CESC patients with durable clinical benefits showed lower risk scores. Thus, we confirmed that the low-risk patients have a better response to CCRT, which provided a potential direction for the chemotherapy management of CESC patients. In summary, our findings suggest that the dsRBP signature could serve as a valuable tool for identifying potential responders to ICIs and CCRT treatment in CESC. By utilizing this signature, we may be able to improve the clinical outcomes of CESC patients by prioritizing therapies.

## Conclusions

To our knowledge, this is the first study to comprehensively investigate the expression profile and prognostic significance of dsRBPs in CESC, utilizing both bulk RNA and single-cell RNA sequencing data. Our study unveiled a distinct and previously uncharacterized expression pattern of dsRBP subtypes in CESC. Furthermore, we identified a unique cluster that exhibited associations with clinical outcomes, genomic characteristics, tumor microenvironment landscape, and immune features in CESC. Additionally, we developed a risk model based on the dsRBP cluster, which demonstrated its potential as a prognostic predictor, which not only provided valuable prognostic information but also aided in determining the potential clinical benefits of chemotherapy and immunotherapy for CESC patients. Our findings highlight the importance of dsRBPs in CESC and shed light on their potential as biomarkers and therapeutic targets in the management of this disease.

### Supplementary Information

**Additional file 1: Figure S1. **Identification of dsRBPs expression feature correlated with HPV infection in CESC samples from TCGA-CESC cohort. Comparison in the ssGSEA scores of each dsRBP subtype in CESC samples classified by HPV status (A), hierarchical HPV call (B), hierarchical HPV clade (C) and HPV integration status (D). dsRBPs: double-stranded RNA-binding proteins; ssGSEA: single sample Gene Set Enrichment Analysis. *p < 0.05; **p < 0.01; ***p < 0.001; ****p < 0.0001; ns: non-significant. **Figure S2.** Tumor immunity related to dsRBPs expression patterns in cervical cancer. Analysis of the correlation between the expression seven dsRBPs subtypes and the level of immune score (A), tumor-infiltrated immune cells (B) and immune checkpoints (C). *p < 0.05; **p < 0.01; ***p < 0.001; ****p < 0.0001. **Figure S3.** Boxplot showed the different expression levels of ADAR and DDR subfamily members among CESC samples with different HPV infection status in the TCGA-CESC cohort. ADAR: adenosine deaminases acting on RNA, DDR: Dicer, Drosha, and Argonautes. *p < 0.05; ns: non-significant. **Figure S4.** The correlation between clinical parameters and different clusters, such as age, T stage, N stage, M stage, and neoplasm disease stage. **Figure S5.** The distribution of HPV-infection patients in different dsRBP clusters. **Figure S6**. The Kaplan-Meier plots of overall survival stratified by age (≤ 60/> 60), T stage (T1–2/T3–4), N stage (N0/N1+), M stage (M0/M1), and neoplasm disease stage (stage I–II/stage III–IV). **Figure S7.** Univariate and multivariate Cox regression analysis of risk score and clinicopathological parameters. **Figure S8.** Comparison the of dsRBPs signature risk scores among CESC patients with different HPV infection status. *p < 0.05; **p < 0.01; ns: non-significant. Difference in the dsRBPs signature risk scores between CESC samples classified by HPV status (A), hierarchical HPV call (B), hierarchical HPV clade (C) and HPV integration status (D). **Figure S9.** Oncoplot of the genomic alterations in hub genes identified in the cervical cancer samples from the TCGA database.

**Additional file 2: Table S1.** 35 dsRBPs colected and applied in current study. **Table S2.** Clinical characteristics of cervical cancer samples within different dsRBP clusters. **Table S3.** List of dsRBP cluster-associated DEGs. **Table S4.** List of prognostic dsRBP cluster-associated DEGs. **Table S5.** List of further achieved prognostic dsRBP cluster-associated DEGs after multistep filtering. **Table S6.** performance of signature in the TCGA-CESC cohort, GSE44001 cohort and CGCI-HTMCP-CC cohort. **Table S7.** Patients information stratified by the signature in the TCGA-CESC cohort. **Table S8.** Clinical feature information of the high- and low-risk group. **Table S9.** Sample information stratified by the signature in the GSE44001 validation cohort. **Table S10.** Sample information stratified by the signature in the CGCI-HTMCP-CC validation cohort. **Table S11.** Differentially expressed genes between Scissor+ and Scissor− cells.

## Data Availability

The datasets generated during and/or analysed during the current study are deposited in the National Genomics Data Center, China National Center for Bioinformation/Beijing Institute of Genomics, Chinese Academy of Sciences repository.
