# Supplementary figures and images for: Characterization of tumor microenvironment and tumor immunology based on the double-stranded RNA-binding protein related genes in cervical cancer
Source: J Transl Med. 2023 Sep 21;21:647. doi: 10.1186/s12967-023-04505-9 (PMC10515034; doi:10.1186/s12967-023-04505-9)

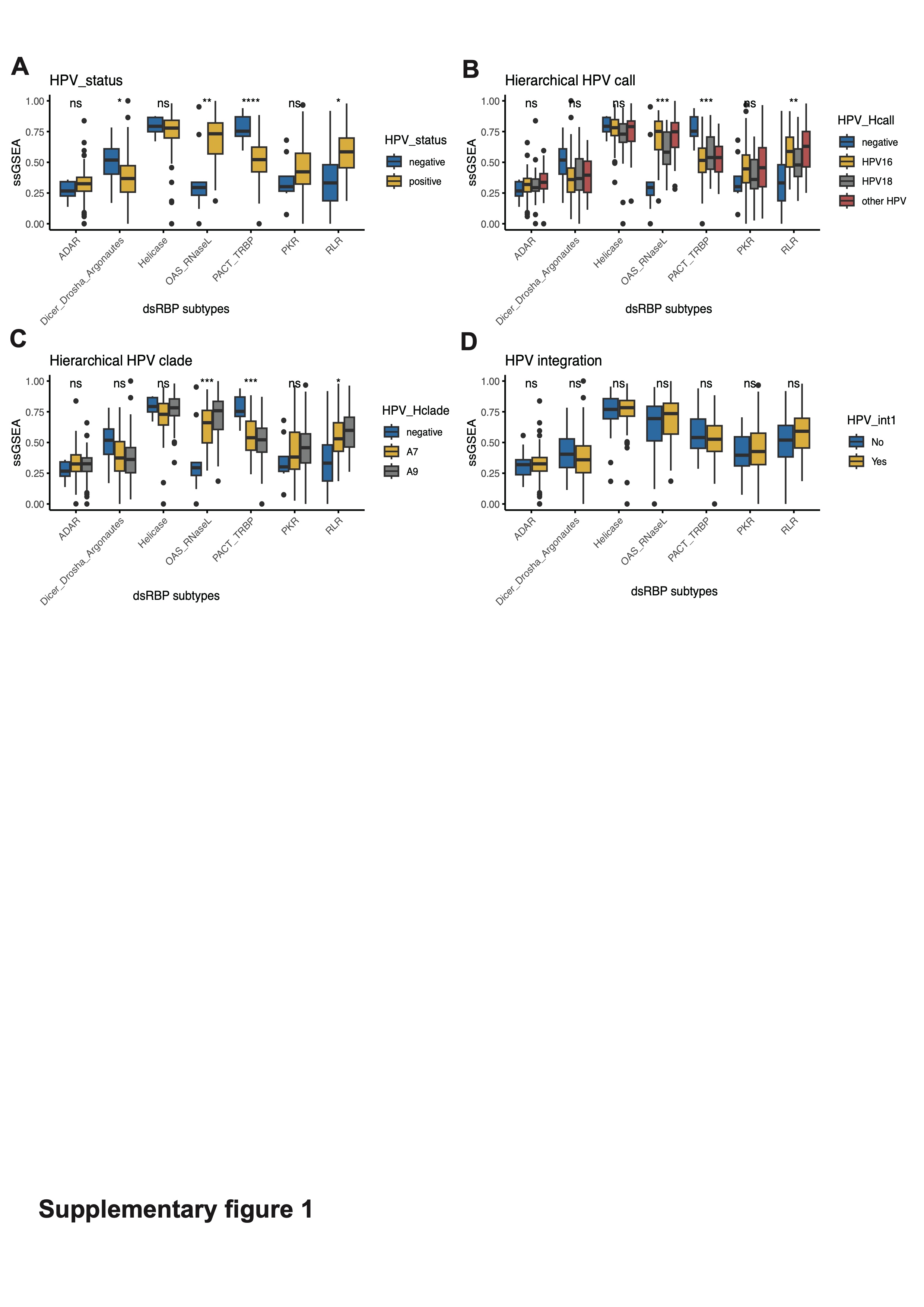

Supplement: Supplementary file 1 — Additional file 1: Figure S1. Identification of dsRBPs expression feature correlated with HPV infection in CESC samples from TCGA-CESC cohort. Comparison in the ssGSEA scores of each dsRBP subtype in CESC samples classified by HPV status (A), hierarchical HPV call (B), hierarchical HPV clade (C) and HPV integration status (D). dsRBPs: double-stranded RNA-binding proteins; ssGSEA: single sample Gene Set Enrichment Analysis. *p < 0.05; **p < 0.01; ***p < 0.001; ****p < 0.0001; ns: non-significant. Figure S2. Tumor immunity related to dsRBPs expression patterns in cervical cancer. Analysis of the correlation between the expression seven dsRBPs subtypes and the level of immune score (A), tumor-infiltrated immune cells (B) and immune checkpoints (C). *p < 0.05; **p < 0.01; ***p < 0.001; ****p < 0.0001. Figure S3. Boxplot showed the different expression levels of ADAR and DDR subfamily members among CESC samples with different HPV infection status in the TCGA-CESC cohort. ADAR: adenosine deaminases acting on RNA, DDR: Dicer, Drosha, and Argonautes. *p < 0.05; ns: non-significant. Figure S4. The correlation between clinical parameters and different clusters, such as age, T stage, N stage, M stage, and neoplasm disease stage. Figure S5. The distribution of HPV-infection patients in different dsRBP clusters. Figure S6. The Kaplan-Meier plots of overall survival stratified by age (≤ 60/> 60), T stage (T1–2/T3–4), N stage (N0/N1+), M stage (M0/M1), and neoplasm disease stage (stage I–II/stage III–IV). Figure S7. Univariate and multivariate Cox regression analysis of risk score and clinicopathological parameters. Figure S8. Comparison the of dsRBPs signature risk scores among CESC patients with different HPV infection status. *p < 0.05; **p < 0.01; ns: non-significant. Difference in the dsRBPs signature risk scores between CESC samples classified by HPV status (A), hierarchical HPV call (B), hierarchical HPV clade (C) and HPV integration status (D). Figure S9. Oncoplot of the g [file 12967_2023_4505_MOESM1_ESM.zip › Figs. S1.jpg]

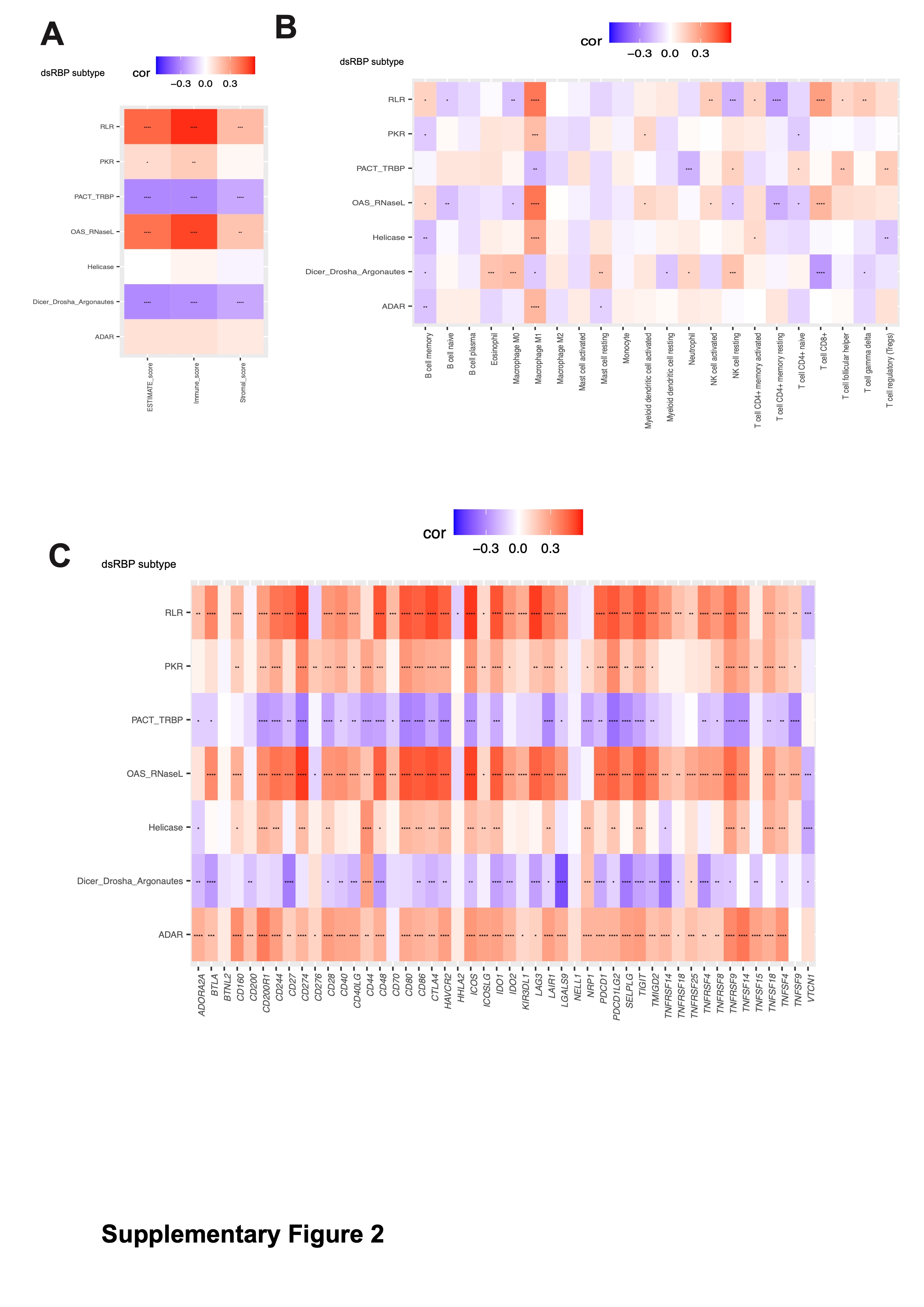

Supplement: Supplementary file 1 — Additional file 1: Figure S1. Identification of dsRBPs expression feature correlated with HPV infection in CESC samples from TCGA-CESC cohort. Comparison in the ssGSEA scores of each dsRBP subtype in CESC samples classified by HPV status (A), hierarchical HPV call (B), hierarchical HPV clade (C) and HPV integration status (D). dsRBPs: double-stranded RNA-binding proteins; ssGSEA: single sample Gene Set Enrichment Analysis. *p < 0.05; **p < 0.01; ***p < 0.001; ****p < 0.0001; ns: non-significant. Figure S2. Tumor immunity related to dsRBPs expression patterns in cervical cancer. Analysis of the correlation between the expression seven dsRBPs subtypes and the level of immune score (A), tumor-infiltrated immune cells (B) and immune checkpoints (C). *p < 0.05; **p < 0.01; ***p < 0.001; ****p < 0.0001. Figure S3. Boxplot showed the different expression levels of ADAR and DDR subfamily members among CESC samples with different HPV infection status in the TCGA-CESC cohort. ADAR: adenosine deaminases acting on RNA, DDR: Dicer, Drosha, and Argonautes. *p < 0.05; ns: non-significant. Figure S4. The correlation between clinical parameters and different clusters, such as age, T stage, N stage, M stage, and neoplasm disease stage. Figure S5. The distribution of HPV-infection patients in different dsRBP clusters. Figure S6. The Kaplan-Meier plots of overall survival stratified by age (≤ 60/> 60), T stage (T1–2/T3–4), N stage (N0/N1+), M stage (M0/M1), and neoplasm disease stage (stage I–II/stage III–IV). Figure S7. Univariate and multivariate Cox regression analysis of risk score and clinicopathological parameters. Figure S8. Comparison the of dsRBPs signature risk scores among CESC patients with different HPV infection status. *p < 0.05; **p < 0.01; ns: non-significant. Difference in the dsRBPs signature risk scores between CESC samples classified by HPV status (A), hierarchical HPV call (B), hierarchical HPV clade (C) and HPV integration status (D). Figure S9. Oncoplot of the g [file 12967_2023_4505_MOESM1_ESM.zip › Figs. S2.jpg]

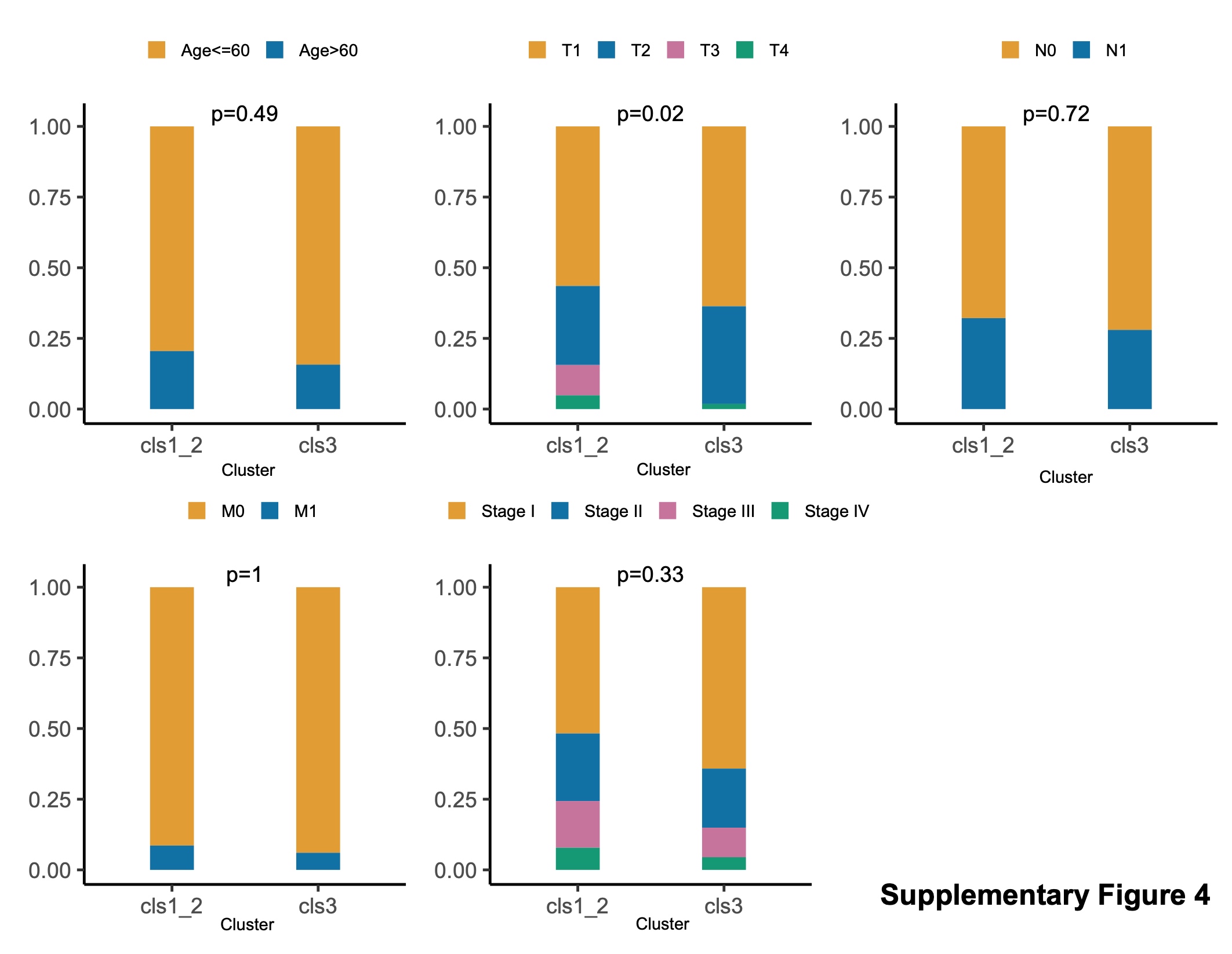

Supplement: Supplementary file 1 — Additional file 1: Figure S1. Identification of dsRBPs expression feature correlated with HPV infection in CESC samples from TCGA-CESC cohort. Comparison in the ssGSEA scores of each dsRBP subtype in CESC samples classified by HPV status (A), hierarchical HPV call (B), hierarchical HPV clade (C) and HPV integration status (D). dsRBPs: double-stranded RNA-binding proteins; ssGSEA: single sample Gene Set Enrichment Analysis. *p < 0.05; **p < 0.01; ***p < 0.001; ****p < 0.0001; ns: non-significant. Figure S2. Tumor immunity related to dsRBPs expression patterns in cervical cancer. Analysis of the correlation between the expression seven dsRBPs subtypes and the level of immune score (A), tumor-infiltrated immune cells (B) and immune checkpoints (C). *p < 0.05; **p < 0.01; ***p < 0.001; ****p < 0.0001. Figure S3. Boxplot showed the different expression levels of ADAR and DDR subfamily members among CESC samples with different HPV infection status in the TCGA-CESC cohort. ADAR: adenosine deaminases acting on RNA, DDR: Dicer, Drosha, and Argonautes. *p < 0.05; ns: non-significant. Figure S4. The correlation between clinical parameters and different clusters, such as age, T stage, N stage, M stage, and neoplasm disease stage. Figure S5. The distribution of HPV-infection patients in different dsRBP clusters. Figure S6. The Kaplan-Meier plots of overall survival stratified by age (≤ 60/> 60), T stage (T1–2/T3–4), N stage (N0/N1+), M stage (M0/M1), and neoplasm disease stage (stage I–II/stage III–IV). Figure S7. Univariate and multivariate Cox regression analysis of risk score and clinicopathological parameters. Figure S8. Comparison the of dsRBPs signature risk scores among CESC patients with different HPV infection status. *p < 0.05; **p < 0.01; ns: non-significant. Difference in the dsRBPs signature risk scores between CESC samples classified by HPV status (A), hierarchical HPV call (B), hierarchical HPV clade (C) and HPV integration status (D). Figure S9. Oncoplot of the g [file 12967_2023_4505_MOESM1_ESM.zip › Figs. S4.jpg]

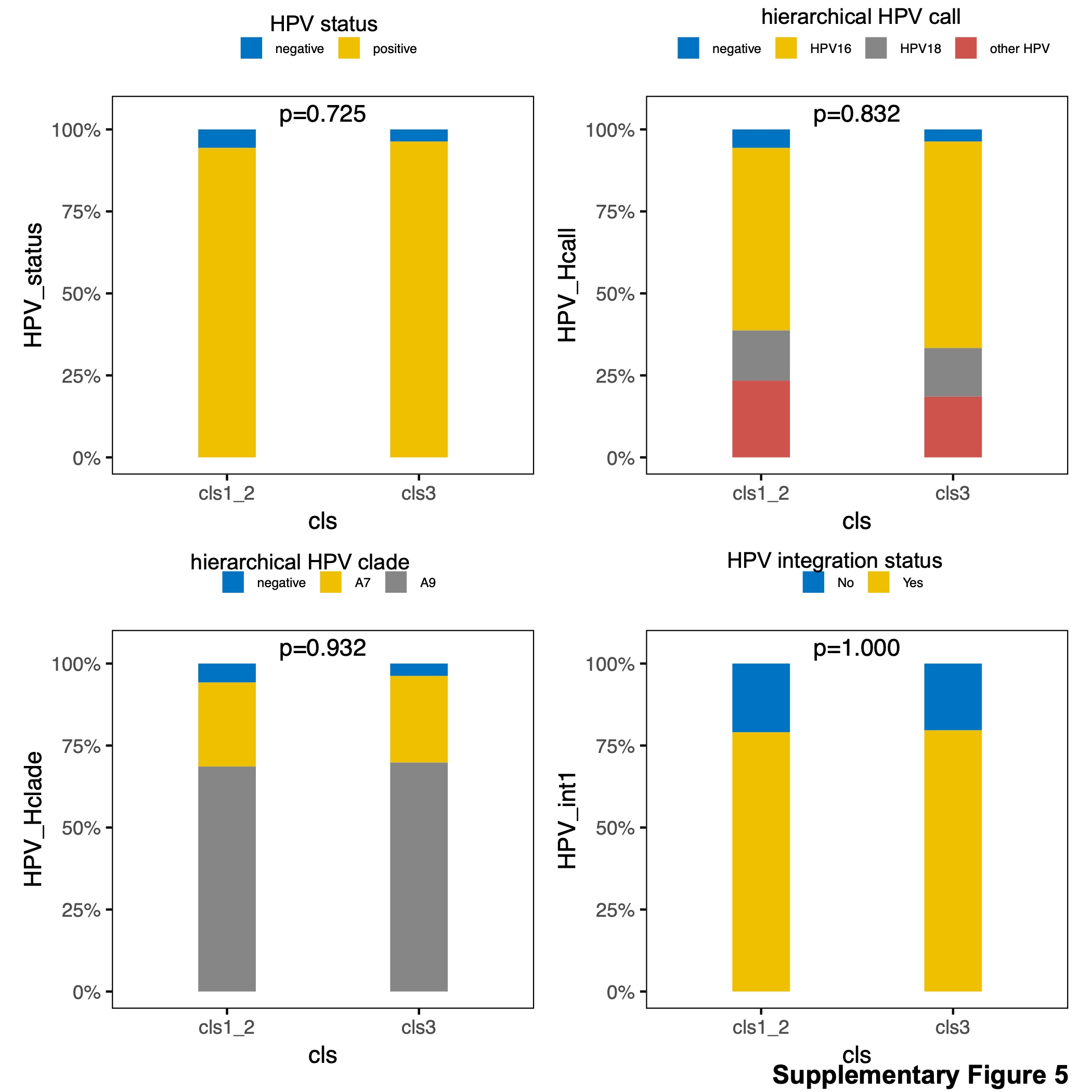

Supplement: Supplementary file 1 — Additional file 1: Figure S1. Identification of dsRBPs expression feature correlated with HPV infection in CESC samples from TCGA-CESC cohort. Comparison in the ssGSEA scores of each dsRBP subtype in CESC samples classified by HPV status (A), hierarchical HPV call (B), hierarchical HPV clade (C) and HPV integration status (D). dsRBPs: double-stranded RNA-binding proteins; ssGSEA: single sample Gene Set Enrichment Analysis. *p < 0.05; **p < 0.01; ***p < 0.001; ****p < 0.0001; ns: non-significant. Figure S2. Tumor immunity related to dsRBPs expression patterns in cervical cancer. Analysis of the correlation between the expression seven dsRBPs subtypes and the level of immune score (A), tumor-infiltrated immune cells (B) and immune checkpoints (C). *p < 0.05; **p < 0.01; ***p < 0.001; ****p < 0.0001. Figure S3. Boxplot showed the different expression levels of ADAR and DDR subfamily members among CESC samples with different HPV infection status in the TCGA-CESC cohort. ADAR: adenosine deaminases acting on RNA, DDR: Dicer, Drosha, and Argonautes. *p < 0.05; ns: non-significant. Figure S4. The correlation between clinical parameters and different clusters, such as age, T stage, N stage, M stage, and neoplasm disease stage. Figure S5. The distribution of HPV-infection patients in different dsRBP clusters. Figure S6. The Kaplan-Meier plots of overall survival stratified by age (≤ 60/> 60), T stage (T1–2/T3–4), N stage (N0/N1+), M stage (M0/M1), and neoplasm disease stage (stage I–II/stage III–IV). Figure S7. Univariate and multivariate Cox regression analysis of risk score and clinicopathological parameters. Figure S8. Comparison the of dsRBPs signature risk scores among CESC patients with different HPV infection status. *p < 0.05; **p < 0.01; ns: non-significant. Difference in the dsRBPs signature risk scores between CESC samples classified by HPV status (A), hierarchical HPV call (B), hierarchical HPV clade (C) and HPV integration status (D). Figure S9. Oncoplot of the g [file 12967_2023_4505_MOESM1_ESM.zip › Figs. S5.jpg]

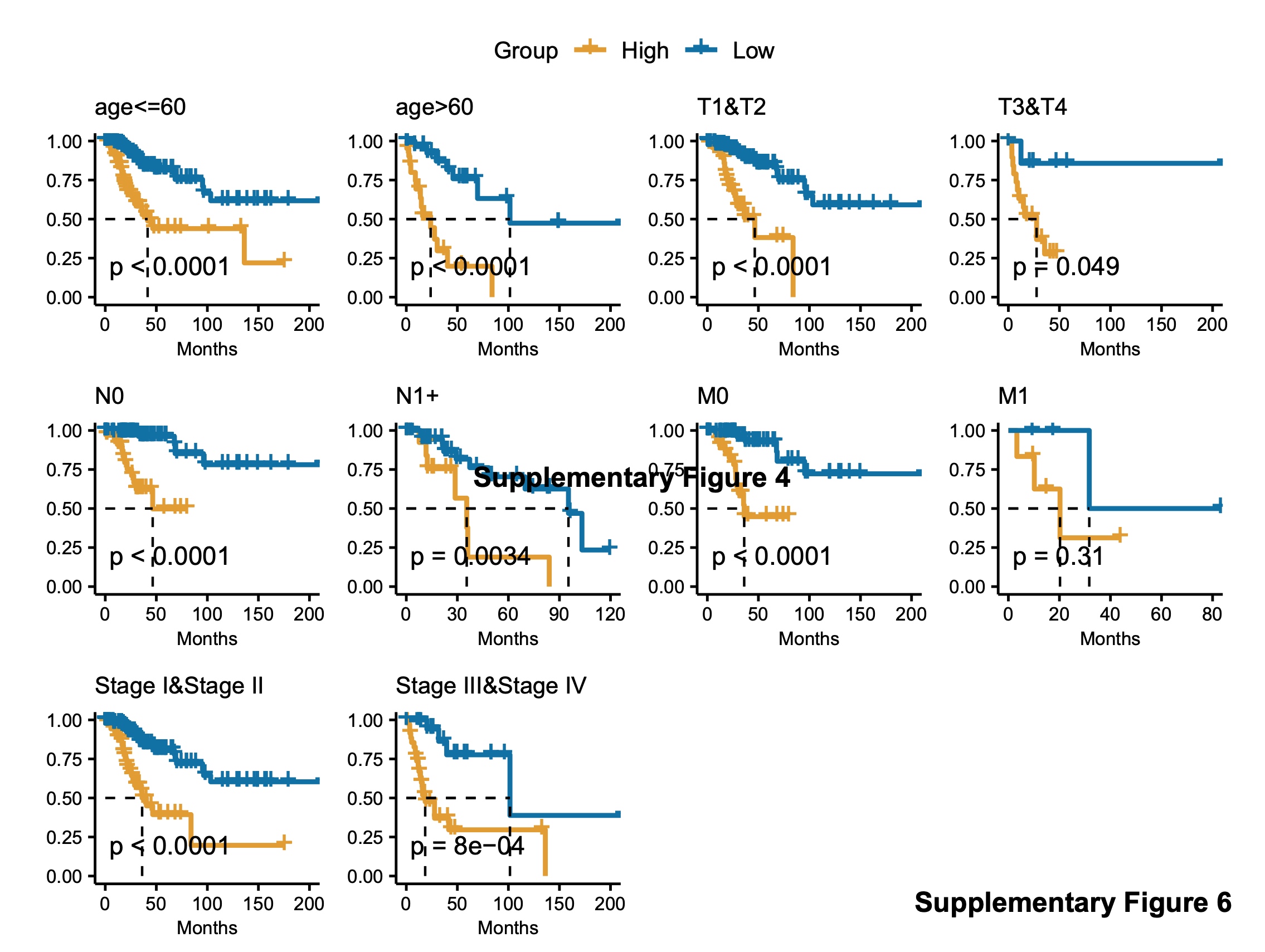

Supplement: Supplementary file 1 — Additional file 1: Figure S1. Identification of dsRBPs expression feature correlated with HPV infection in CESC samples from TCGA-CESC cohort. Comparison in the ssGSEA scores of each dsRBP subtype in CESC samples classified by HPV status (A), hierarchical HPV call (B), hierarchical HPV clade (C) and HPV integration status (D). dsRBPs: double-stranded RNA-binding proteins; ssGSEA: single sample Gene Set Enrichment Analysis. *p < 0.05; **p < 0.01; ***p < 0.001; ****p < 0.0001; ns: non-significant. Figure S2. Tumor immunity related to dsRBPs expression patterns in cervical cancer. Analysis of the correlation between the expression seven dsRBPs subtypes and the level of immune score (A), tumor-infiltrated immune cells (B) and immune checkpoints (C). *p < 0.05; **p < 0.01; ***p < 0.001; ****p < 0.0001. Figure S3. Boxplot showed the different expression levels of ADAR and DDR subfamily members among CESC samples with different HPV infection status in the TCGA-CESC cohort. ADAR: adenosine deaminases acting on RNA, DDR: Dicer, Drosha, and Argonautes. *p < 0.05; ns: non-significant. Figure S4. The correlation between clinical parameters and different clusters, such as age, T stage, N stage, M stage, and neoplasm disease stage. Figure S5. The distribution of HPV-infection patients in different dsRBP clusters. Figure S6. The Kaplan-Meier plots of overall survival stratified by age (≤ 60/> 60), T stage (T1–2/T3–4), N stage (N0/N1+), M stage (M0/M1), and neoplasm disease stage (stage I–II/stage III–IV). Figure S7. Univariate and multivariate Cox regression analysis of risk score and clinicopathological parameters. Figure S8. Comparison the of dsRBPs signature risk scores among CESC patients with different HPV infection status. *p < 0.05; **p < 0.01; ns: non-significant. Difference in the dsRBPs signature risk scores between CESC samples classified by HPV status (A), hierarchical HPV call (B), hierarchical HPV clade (C) and HPV integration status (D). Figure S9. Oncoplot of the g [file 12967_2023_4505_MOESM1_ESM.zip › Figs. S6.jpg]

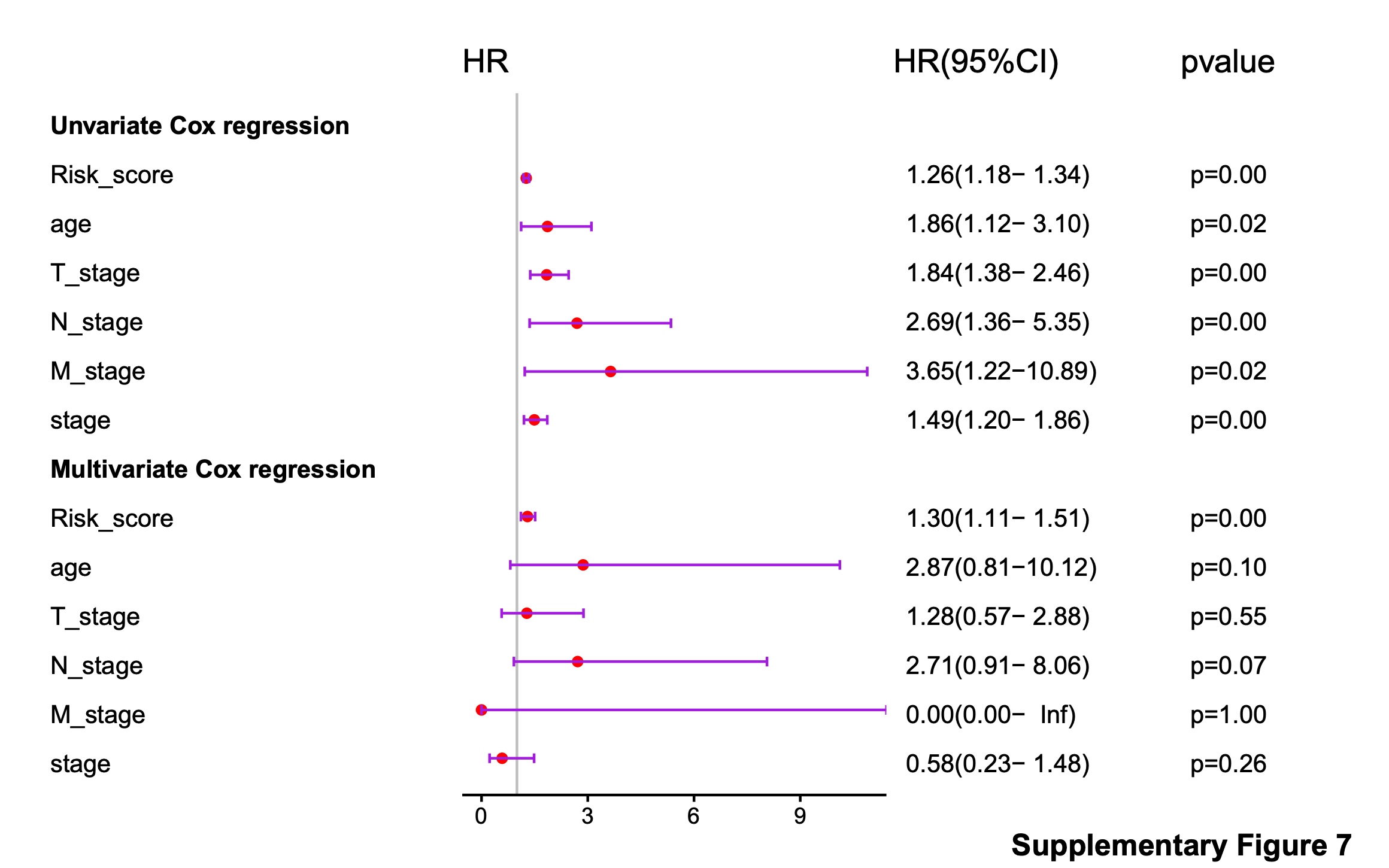

Supplement: Supplementary file 1 — Additional file 1: Figure S1. Identification of dsRBPs expression feature correlated with HPV infection in CESC samples from TCGA-CESC cohort. Comparison in the ssGSEA scores of each dsRBP subtype in CESC samples classified by HPV status (A), hierarchical HPV call (B), hierarchical HPV clade (C) and HPV integration status (D). dsRBPs: double-stranded RNA-binding proteins; ssGSEA: single sample Gene Set Enrichment Analysis. *p < 0.05; **p < 0.01; ***p < 0.001; ****p < 0.0001; ns: non-significant. Figure S2. Tumor immunity related to dsRBPs expression patterns in cervical cancer. Analysis of the correlation between the expression seven dsRBPs subtypes and the level of immune score (A), tumor-infiltrated immune cells (B) and immune checkpoints (C). *p < 0.05; **p < 0.01; ***p < 0.001; ****p < 0.0001. Figure S3. Boxplot showed the different expression levels of ADAR and DDR subfamily members among CESC samples with different HPV infection status in the TCGA-CESC cohort. ADAR: adenosine deaminases acting on RNA, DDR: Dicer, Drosha, and Argonautes. *p < 0.05; ns: non-significant. Figure S4. The correlation between clinical parameters and different clusters, such as age, T stage, N stage, M stage, and neoplasm disease stage. Figure S5. The distribution of HPV-infection patients in different dsRBP clusters. Figure S6. The Kaplan-Meier plots of overall survival stratified by age (≤ 60/> 60), T stage (T1–2/T3–4), N stage (N0/N1+), M stage (M0/M1), and neoplasm disease stage (stage I–II/stage III–IV). Figure S7. Univariate and multivariate Cox regression analysis of risk score and clinicopathological parameters. Figure S8. Comparison the of dsRBPs signature risk scores among CESC patients with different HPV infection status. *p < 0.05; **p < 0.01; ns: non-significant. Difference in the dsRBPs signature risk scores between CESC samples classified by HPV status (A), hierarchical HPV call (B), hierarchical HPV clade (C) and HPV integration status (D). Figure S9. Oncoplot of the g [file 12967_2023_4505_MOESM1_ESM.zip › Figs. S7.jpg]

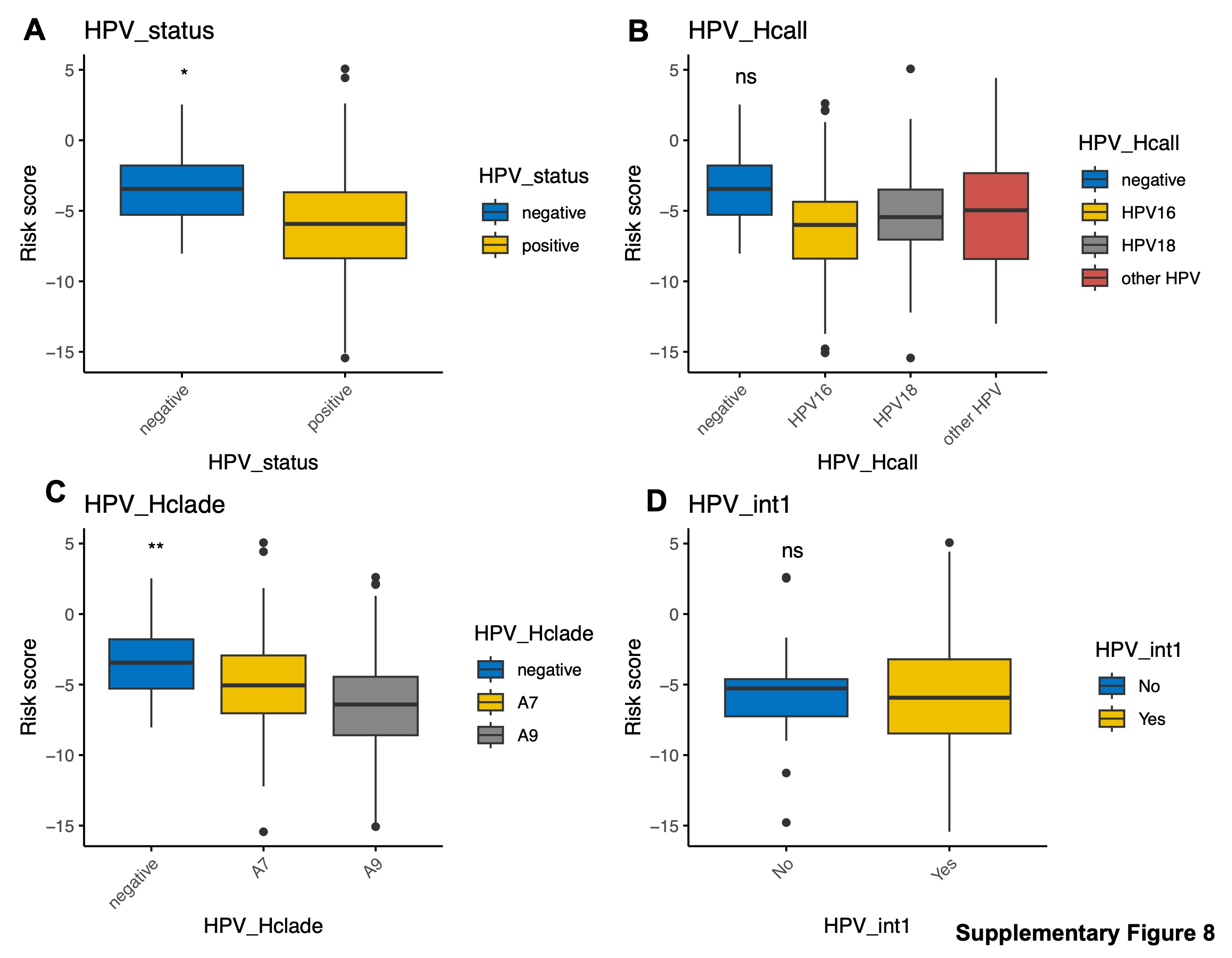

Supplement: Supplementary file 1 — Additional file 1: Figure S1. Identification of dsRBPs expression feature correlated with HPV infection in CESC samples from TCGA-CESC cohort. Comparison in the ssGSEA scores of each dsRBP subtype in CESC samples classified by HPV status (A), hierarchical HPV call (B), hierarchical HPV clade (C) and HPV integration status (D). dsRBPs: double-stranded RNA-binding proteins; ssGSEA: single sample Gene Set Enrichment Analysis. *p < 0.05; **p < 0.01; ***p < 0.001; ****p < 0.0001; ns: non-significant. Figure S2. Tumor immunity related to dsRBPs expression patterns in cervical cancer. Analysis of the correlation between the expression seven dsRBPs subtypes and the level of immune score (A), tumor-infiltrated immune cells (B) and immune checkpoints (C). *p < 0.05; **p < 0.01; ***p < 0.001; ****p < 0.0001. Figure S3. Boxplot showed the different expression levels of ADAR and DDR subfamily members among CESC samples with different HPV infection status in the TCGA-CESC cohort. ADAR: adenosine deaminases acting on RNA, DDR: Dicer, Drosha, and Argonautes. *p < 0.05; ns: non-significant. Figure S4. The correlation between clinical parameters and different clusters, such as age, T stage, N stage, M stage, and neoplasm disease stage. Figure S5. The distribution of HPV-infection patients in different dsRBP clusters. Figure S6. The Kaplan-Meier plots of overall survival stratified by age (≤ 60/> 60), T stage (T1–2/T3–4), N stage (N0/N1+), M stage (M0/M1), and neoplasm disease stage (stage I–II/stage III–IV). Figure S7. Univariate and multivariate Cox regression analysis of risk score and clinicopathological parameters. Figure S8. Comparison the of dsRBPs signature risk scores among CESC patients with different HPV infection status. *p < 0.05; **p < 0.01; ns: non-significant. Difference in the dsRBPs signature risk scores between CESC samples classified by HPV status (A), hierarchical HPV call (B), hierarchical HPV clade (C) and HPV integration status (D). Figure S9. Oncoplot of the g [file 12967_2023_4505_MOESM1_ESM.zip › Figs. S8.jpg]

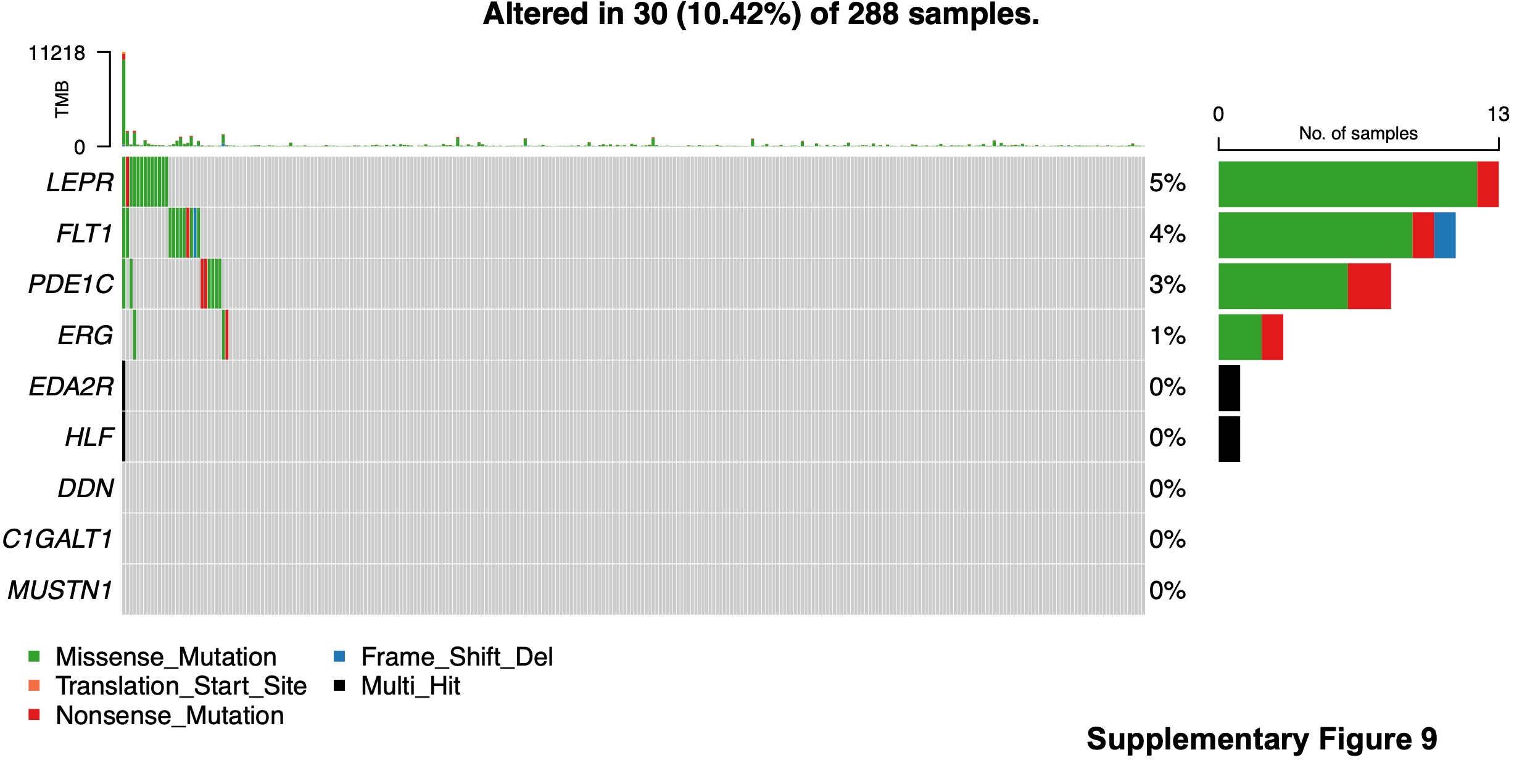

Supplement: Supplementary file 1 — Additional file 1: Figure S1. Identification of dsRBPs expression feature correlated with HPV infection in CESC samples from TCGA-CESC cohort. Comparison in the ssGSEA scores of each dsRBP subtype in CESC samples classified by HPV status (A), hierarchical HPV call (B), hierarchical HPV clade (C) and HPV integration status (D). dsRBPs: double-stranded RNA-binding proteins; ssGSEA: single sample Gene Set Enrichment Analysis. *p < 0.05; **p < 0.01; ***p < 0.001; ****p < 0.0001; ns: non-significant. Figure S2. Tumor immunity related to dsRBPs expression patterns in cervical cancer. Analysis of the correlation between the expression seven dsRBPs subtypes and the level of immune score (A), tumor-infiltrated immune cells (B) and immune checkpoints (C). *p < 0.05; **p < 0.01; ***p < 0.001; ****p < 0.0001. Figure S3. Boxplot showed the different expression levels of ADAR and DDR subfamily members among CESC samples with different HPV infection status in the TCGA-CESC cohort. ADAR: adenosine deaminases acting on RNA, DDR: Dicer, Drosha, and Argonautes. *p < 0.05; ns: non-significant. Figure S4. The correlation between clinical parameters and different clusters, such as age, T stage, N stage, M stage, and neoplasm disease stage. Figure S5. The distribution of HPV-infection patients in different dsRBP clusters. Figure S6. The Kaplan-Meier plots of overall survival stratified by age (≤ 60/> 60), T stage (T1–2/T3–4), N stage (N0/N1+), M stage (M0/M1), and neoplasm disease stage (stage I–II/stage III–IV). Figure S7. Univariate and multivariate Cox regression analysis of risk score and clinicopathological parameters. Figure S8. Comparison the of dsRBPs signature risk scores among CESC patients with different HPV infection status. *p < 0.05; **p < 0.01; ns: non-significant. Difference in the dsRBPs signature risk scores between CESC samples classified by HPV status (A), hierarchical HPV call (B), hierarchical HPV clade (C) and HPV integration status (D). Figure S9. Oncoplot of the g [file 12967_2023_4505_MOESM1_ESM.zip › Figs. S9.jpg]

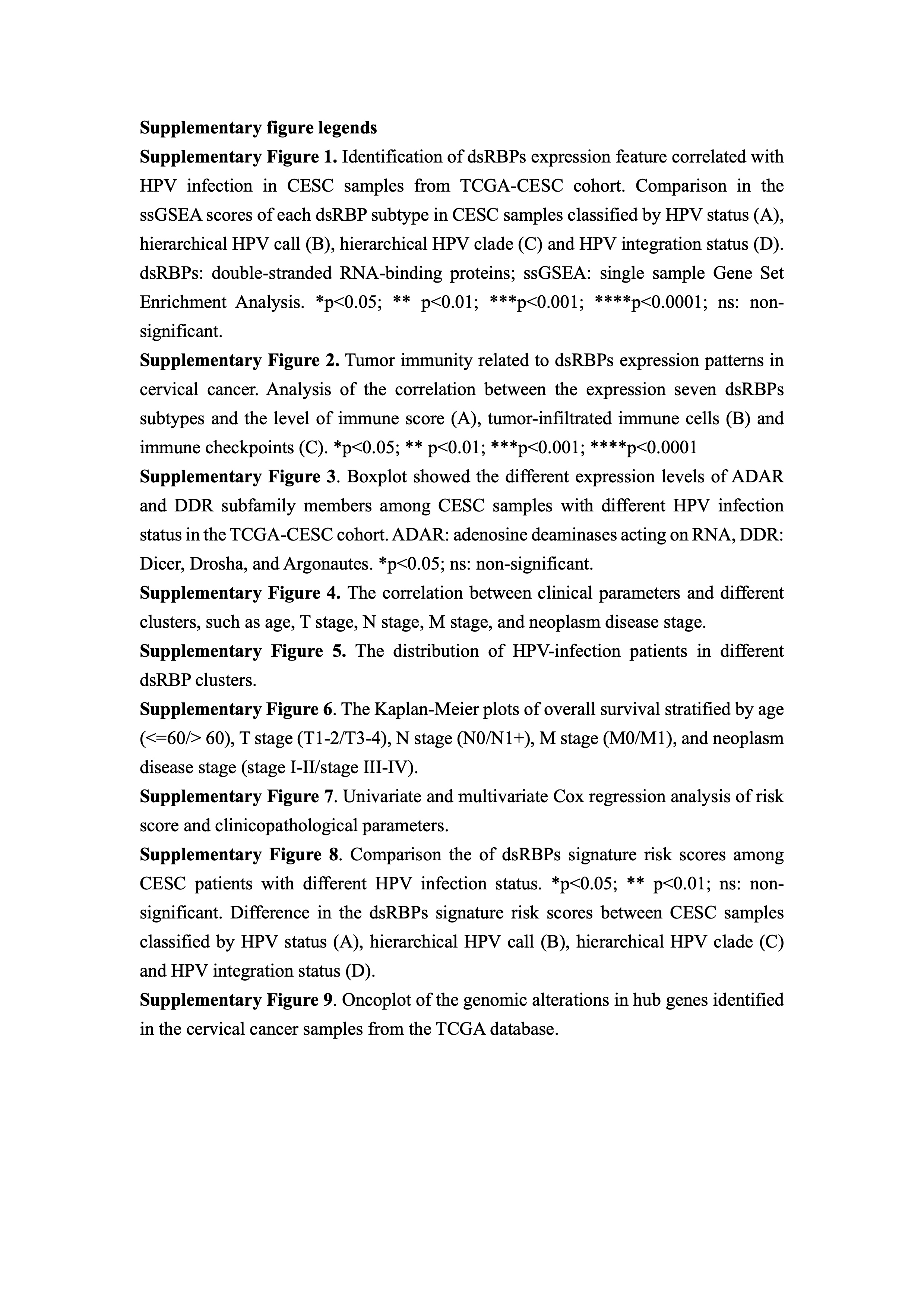

Supplement: Supplementary file 1 — Additional file 1: Figure S1. Identification of dsRBPs expression feature correlated with HPV infection in CESC samples from TCGA-CESC cohort. Comparison in the ssGSEA scores of each dsRBP subtype in CESC samples classified by HPV status (A), hierarchical HPV call (B), hierarchical HPV clade (C) and HPV integration status (D). dsRBPs: double-stranded RNA-binding proteins; ssGSEA: single sample Gene Set Enrichment Analysis. *p < 0.05; **p < 0.01; ***p < 0.001; ****p < 0.0001; ns: non-significant. Figure S2. Tumor immunity related to dsRBPs expression patterns in cervical cancer. Analysis of the correlation between the expression seven dsRBPs subtypes and the level of immune score (A), tumor-infiltrated immune cells (B) and immune checkpoints (C). *p < 0.05; **p < 0.01; ***p < 0.001; ****p < 0.0001. Figure S3. Boxplot showed the different expression levels of ADAR and DDR subfamily members among CESC samples with different HPV infection status in the TCGA-CESC cohort. ADAR: adenosine deaminases acting on RNA, DDR: Dicer, Drosha, and Argonautes. *p < 0.05; ns: non-significant. Figure S4. The correlation between clinical parameters and different clusters, such as age, T stage, N stage, M stage, and neoplasm disease stage. Figure S5. The distribution of HPV-infection patients in different dsRBP clusters. Figure S6. The Kaplan-Meier plots of overall survival stratified by age (≤ 60/> 60), T stage (T1–2/T3–4), N stage (N0/N1+), M stage (M0/M1), and neoplasm disease stage (stage I–II/stage III–IV). Figure S7. Univariate and multivariate Cox regression analysis of risk score and clinicopathological parameters. Figure S8. Comparison the of dsRBPs signature risk scores among CESC patients with different HPV infection status. *p < 0.05; **p < 0.01; ns: non-significant. Difference in the dsRBPs signature risk scores between CESC samples classified by HPV status (A), hierarchical HPV call (B), hierarchical HPV clade (C) and HPV integration status (D). Figure S9. Oncoplot of the g [file 12967_2023_4505_MOESM1_ESM.zip › Figure legends for S1-9.jpg]

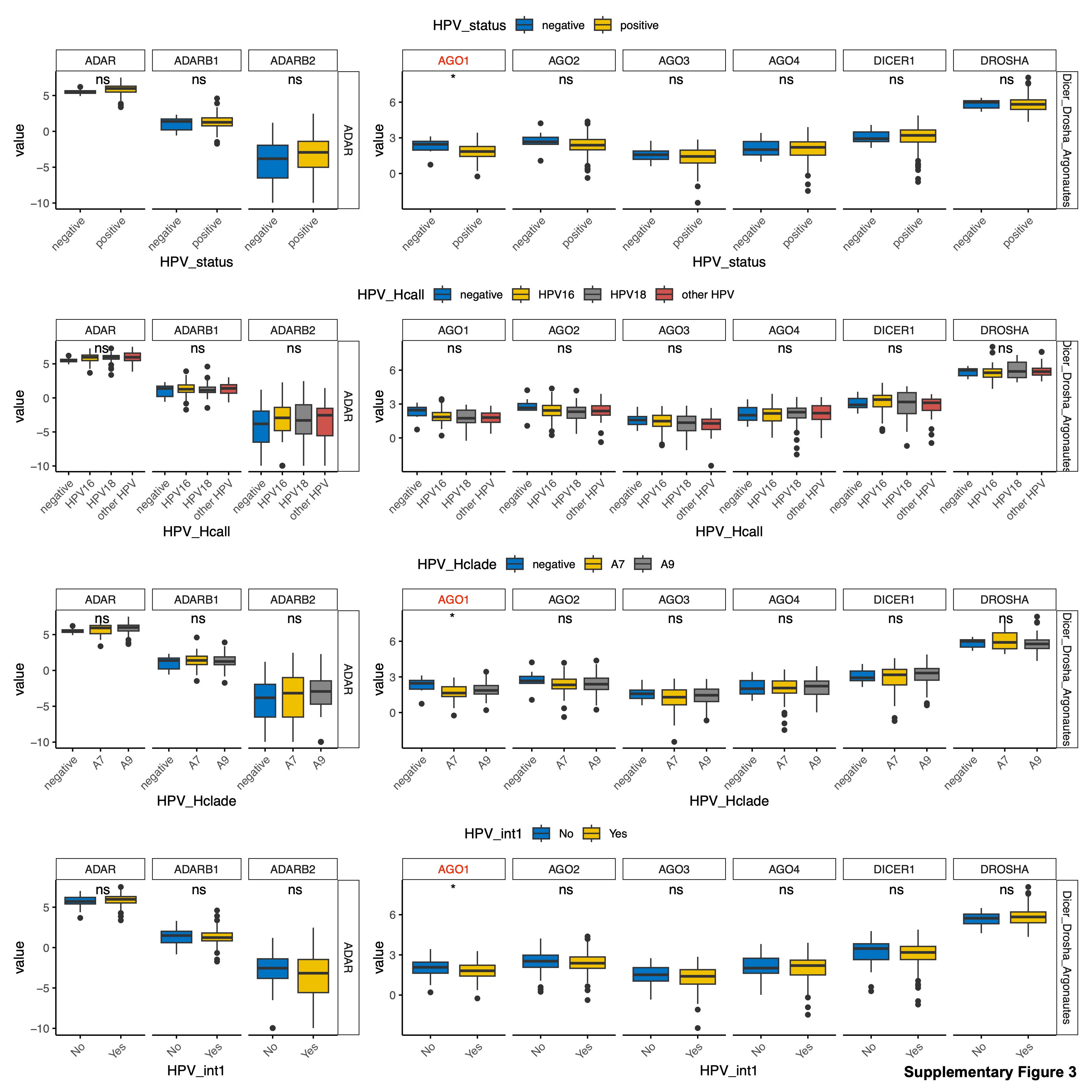

Supplement: Supplementary file 1 — Additional file 1: Figure S1. Identification of dsRBPs expression feature correlated with HPV infection in CESC samples from TCGA-CESC cohort. Comparison in the ssGSEA scores of each dsRBP subtype in CESC samples classified by HPV status (A), hierarchical HPV call (B), hierarchical HPV clade (C) and HPV integration status (D). dsRBPs: double-stranded RNA-binding proteins; ssGSEA: single sample Gene Set Enrichment Analysis. *p < 0.05; **p < 0.01; ***p < 0.001; ****p < 0.0001; ns: non-significant. Figure S2. Tumor immunity related to dsRBPs expression patterns in cervical cancer. Analysis of the correlation between the expression seven dsRBPs subtypes and the level of immune score (A), tumor-infiltrated immune cells (B) and immune checkpoints (C). *p < 0.05; **p < 0.01; ***p < 0.001; ****p < 0.0001. Figure S3. Boxplot showed the different expression levels of ADAR and DDR subfamily members among CESC samples with different HPV infection status in the TCGA-CESC cohort. ADAR: adenosine deaminases acting on RNA, DDR: Dicer, Drosha, and Argonautes. *p < 0.05; ns: non-significant. Figure S4. The correlation between clinical parameters and different clusters, such as age, T stage, N stage, M stage, and neoplasm disease stage. Figure S5. The distribution of HPV-infection patients in different dsRBP clusters. Figure S6. The Kaplan-Meier plots of overall survival stratified by age (≤ 60/> 60), T stage (T1–2/T3–4), N stage (N0/N1+), M stage (M0/M1), and neoplasm disease stage (stage I–II/stage III–IV). Figure S7. Univariate and multivariate Cox regression analysis of risk score and clinicopathological parameters. Figure S8. Comparison the of dsRBPs signature risk scores among CESC patients with different HPV infection status. *p < 0.05; **p < 0.01; ns: non-significant. Difference in the dsRBPs signature risk scores between CESC samples classified by HPV status (A), hierarchical HPV call (B), hierarchical HPV clade (C) and HPV integration status (D). Figure S9. Oncoplot of the g [file 12967_2023_4505_MOESM1_ESM.zip › page 4.jpg]
